# Reconstructing population dynamics of a threatened marine mammal using multiple data sets

**DOI:** 10.1038/s41598-021-81478-z

**Published:** 2021-01-29

**Authors:** Jeffrey A. Hostetler, Julien Martin, Michael Kosempa, Holly H. Edwards, Kari A. Rood, Sheri L. Barton, Michael C. Runge

**Affiliations:** 1 Fish and Wildlife Research Institute, Florida Fish and Wildlife Conservation Commission, St. Petersburg, FL 33701 USA; 2 Wetland and Aquatic Research Center, U.S. Geological Survey, Gainesville, FL 32653 USA; 3 U.S. Geological Survey, St. Petersburg Coastal and Marine Science Center, St. Petersburg, FL 33701 USA; 4 Mote Marine Laboratory, 1600 Ken Thompson Parkway, Sarasota, FL 34236 USA; 5 Patuxent Wildlife Research Center, U.S. Geological Survey, 12100 Beech Forest Road, Laurel, MD 20708 USA; 6 Present Address: Patuxent Wildlife Research Center, U.S. Fish and Wildlife Service, Laurel, MD 20708 USA

**Keywords:** Ecological modelling, Population dynamics

## Abstract

Models of marine mammal population dynamics have been used extensively to predict abundance. A less common application of these models is to reconstruct historical population dynamics, filling in gaps in observation data by integrating information from multiple sources. We developed an integrated population model for the Florida manatee (*Trichechus manatus latirostris*) to reconstruct its population dynamics in the southwest region of the state over the past 20 years. Our model improved precision of key parameter estimates and permitted inference on poorly known parameters. Population growth was slow (averaging 1.02; 95% credible interval 1.01–1.03) but not steady, and an unusual mortality event in 2013 led to an estimated net loss of 332 (217–466) manatees. Our analyses showed that precise estimates of abundance could be derived from estimates of vital rates and a few input estimates of abundance, which may mean costly surveys to estimate abundance don’t need to be conducted as frequently. Our study also shows that retrospective analyses can be useful to: (1) model the transient dynamics of age distribution; (2) assess and communicate the conservation status of wild populations; and (3) improve our understanding of environmental effects on population dynamics and thus enhance our ability to forecast.

## Introduction

Models of population dynamics are used extensively in ecology and conservation. Applications include predator–prey interactions, decision analyses, and ecological forecast analyses for conservation such as population viability analyses^1–3^. Models of population dynamics are not restricted to the modeling of abundance; they can also be used to model the dynamics of communities or the occupancy status of sites^4–7^. Population viability analyses are a special case of population projection models that focus on the persistence of populations and species, and have been used by many natural-resource-management agencies to assess population status and evaluate threats^1–3^.

Population models for conservation are often used to (1) assess a population’s conservation status; (2) diagnose possible threats; (3) predict the effects of threats or management actions; and (4) identify the best possible actions for achieving management objectives^3,8^. For status assessment, several organizations (e.g., International Union for Conservation of Nature [IUCN]) and natural-resource-management agencies (e.g., U.S. Fish and Wildlife Service [USFWS], National Oceanic and Atmospheric Administration [NOAA]) use quantitative criteria to classify species. For example, the IUCN uses thresholds of percent of decline to classify species into vulnerability categories. Several quantitative criteria are also embedded into the regulatory framework established by the U.S. government. For instance, the computation of the potential biological removal established under the Marine Mammal Protection Act (16 USC 1362[13]) is based on measures of population growth rates and minimum population size. Although the Endangered Species Act (ESA; 16 USC 1531 et seq.) does not have well defined criteria for classification, quantitative measures such as population growth rate, probability of quasiextinction, and minimum population size are commonly used in recovery plans for threatened and endangered species.

Most projection models that have been used for population assessment are not designed to look at historical trends and changes in abundance over short time horizons; instead, these analyses often focus on long-term changes (sometimes by as much as 150 years). Although the effects of environmental perturbations are often considered in a general fashion, the impact of year-specific events is typically not assessed with population viability analyses or other projection models. For instance, Beissinger^9^ and Martin et al.^10^ examined how an increase in drought frequency would affect the population growth rate of snail kites. Similarly, Runge et al.^11^, considered the effect of red tide (*Karenia brevis*) and extreme cold events on Florida manatee (*Trichechus manatus latirostris*) population dynamics. Hostetler et al.^12^ investigated the impact of genetic restoration on the population dynamics of the Florida panther and even ran retrospective and counterfactual analyses. But these analyses did not consider the effect of specific historical disturbances on these populations. For example, there was an unusually cold winter in Florida in 2010: Can we estimate the effect of cold mortality on manatee (or other cold-sensitive populations) abundance in that year?

Construction of prospective analyses often reveals the need for estimates of parameters for which there are few data. In these cases, some sort of expert judgment process is usually used to obtain such estimates. The prospective modeling itself, however, can be sensitive to the missing parameters. Therefore, obtaining empirical estimates of those parameters is often a key goal.

Retrospective population models, i.e., analyses that focus on historical changes in population dynamics, can be used to fill in many of the limitations of prospective models for conservation management and research. They provide insight on historical and current population abundances and realized population growth rates, both of which can be considered in species recovery criteria. They can be used to estimate the effects of specific historical disturbance events on populations and to diagnose both natural and anthropogenic threats. They are also often useful for filling in estimates for parameters with few direct data, such as stage structure or juvenile survival, which can be key to accuracy in population forecasts.

Retrospective analyses can be conducted using a number of methods. Interest is growing in the application of integrated population models (IPMs)^13,14^. IPMs are a relatively new, but rapidly advancing, modeling technique that have been applied to a range of species of conservation concern, including marine mammals^15,16^. A benefit of IPMs is that they facilitate incorporation of multiple sources of information. By simultaneously estimating vital rates and abundance from complementary data streams, IPMs can reduce bias and improve precision of parameter estimates^17^. Many IPMs have been used to estimate latent parameters for which few or no direct data were available and that otherwise would have been inestimable^13,18^. These advantages make IPMs particularly advantageous for providing parameter estimates for population forecast models, sometimes serving as the forecast models themselves^19^. For the same reasons, IPMs are also particularly well suited for evaluating the allocation of monitoring efforts. Although these models are promising, limitations include difficulty in determining whether the model is identifiable, lack of independence of data sets that are being combined, bias from incompatible model components, sensitivity to prior probability distributions on the parameters, and model convergence issues^20^.

Simulation-based hindcast projection models, in which random values are drawn from estimated population-parameter distributions to help fill in missing population parameters, are generally not used for inference directly from data but are often more easily implemented than IPMs. These models can also be used to infer historical changes in population dynamics (or even community dynamics)^21^. This information can be useful in assessing the effect of key environmental events on abundance or other state variables (e.g., occupancy) that present in historical time series. Historically, simulation-based hindcasting of animal populations has received more attention from fishery researchers than from wildlife ecology researchers (see virtual population analysis^22^). Some recent studies have looked at hindcasting of plant communities using predictive models, but such approaches can be challenging, especially when the data do not satisfy the detailed balance condition for time reversibility within Markovian systems^21^.

The Florida manatee was recently reclassified from endangered to threatened by the USFWS under the ESA (82 FR 16668); manatees are also protected under the U.S. Marine Mammal Protection Act. Understanding how the manatee population changes over time and responds to threats (both immediate and long-term) is important for USFWS and Florida Fish and Wildlife Conservation Commission (FWC) managers. A population viability analysis (hereafter referred to as the Core Biological Model, or CBM) has been developed to project manatee abundance, estimate probability of quasiextinction, and evaluate long-term threats (e.g., 100 years)^23^. The CBM is an important tool for managers and policy makers in assessing the status of the manatee population. The CBM was not, however, designed to quantify the effect of specific historical mortality events (e.g., red tide event of 2013 or extreme cold events of 2010); it is also not well suited for making inference about historic trends. Calls for integrating multiple data sources to improve estimates of Florida manatee population parameters date to at least 2004, when Dan Goodman^24^ suggested integrating mark–resight and carcass-recovery data in an extended Jolly-Seber statistical design.

**Figure 1:**
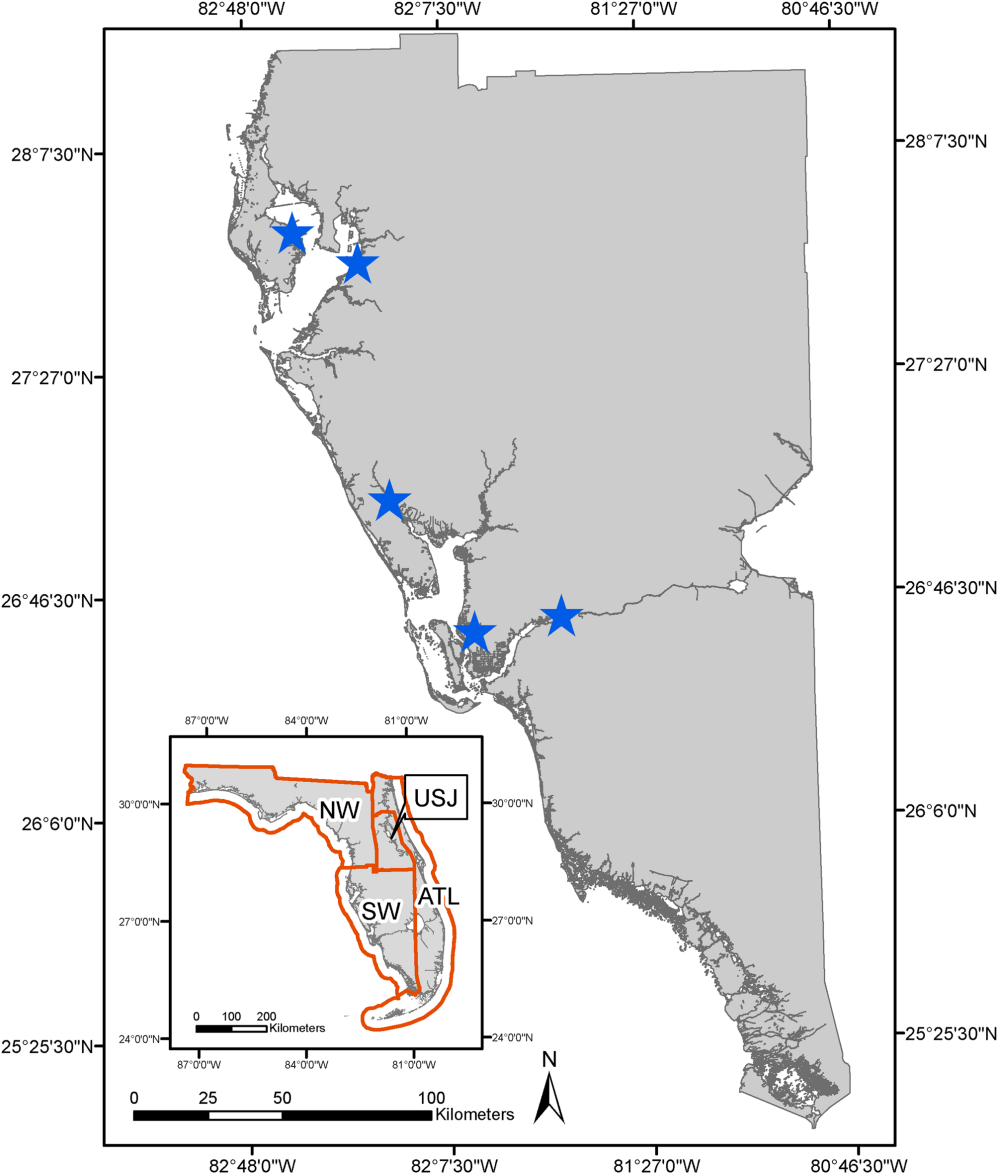
Map of southwest Florida, location of the study population. Florida is divided into four manatee regions or management units (see inset): northwest (NW), southwest (SW), Atlantic coast (ATL), and Upper St. Johns River (USJ). Stars on the map indicate primary locations at which photo-identification data were collected (all are manatee warm-water aggregation sites). Other data streams used in this analysis (carcass data and abundance surveys) were collected throughout potential manatee habitat in southwest Florida and are not pictured. Map generated in ArcMap 10.3 (https://www.esri.com).

**Figure 2:**
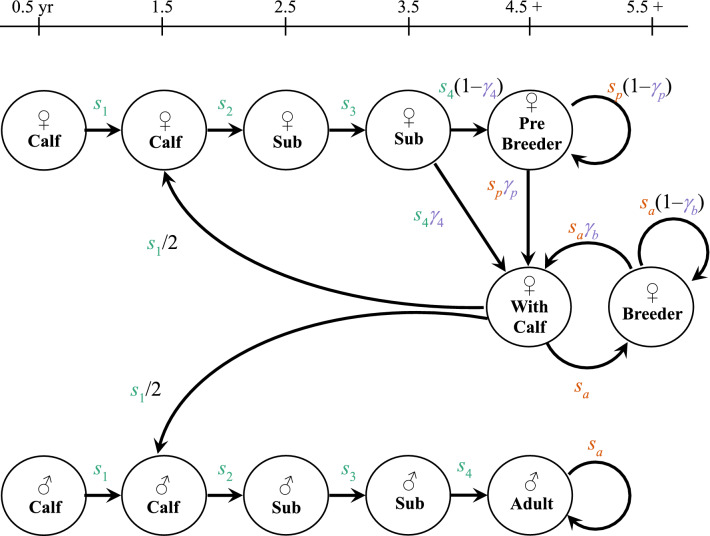
Life cycle diagram of Florida manatees, adapted from Runge et al.^11,23^ Stage-specific survival (*s*) and reproductive (*γ*) probabilities govern the transitions between stages. Females (♀) and males (♂) are both modeled. Calves enter the population model at age 1.5; until that age they are tracked with their mother (0.5-year-old calves are shown in the diagram for completeness). Color indicates the primary sources of information for parameters within the integrated population model: the parameters in green (*s*_1_–*s*_4_) are informed primarily by carcass data and surrogate mortality ratio estimates; the parameters in orange (*s*_*p*_ and *s*_*a*_) are informed primarily by carcass data and mark–recapture survival estimates; and the parameters in purple (*γ*_4_, *γ*_*p*_, and *γ*_*b*_) are informed primarily by mark–recapture reproductive estimates. Other sources of information, including abundance estimates, informed all parameters.

In this paper we present the results of retrospective analyses for Florida manatees in southwest Florida (Fig. 1) that combine two empirical estimates of region-specific abundance from aerial surveys in 2011 and 2016 with carcass-recovery data (1997–2015; *n* = 2711 carcasses) and photo-identification estimates of adult survival and female breeding probabilities (1997–2015). We show how such analyses can be useful (1) for inferring historical trends in abundance, improving our understanding of population dynamics, and therefore enhancing our ability to forecast; (2) for estimating other missing parameters such as recovery probabilities and survival probabilities for calves and subadults; (3) for improving the precision of some earlier population estimates; (4) for modeling the transient dynamics of stage distribution (Fig. 2), which can be important to some populations; (5) for assisting effort allocation of monitoring programs; and (6) for informing management decisions.

## Results

**Figure 3:**
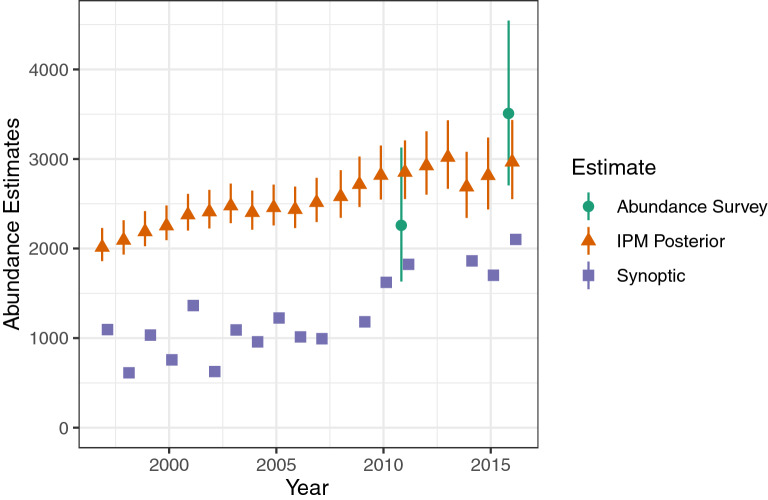
Annual abundance estimates from IPM, 1997–2016 (IPM posterior) and the two aerial abundance surveys. Symbols indicate medians; line segments indicate 95% credible intervals. Synoptic survey counts are shown for reference.

**Figure 4:**
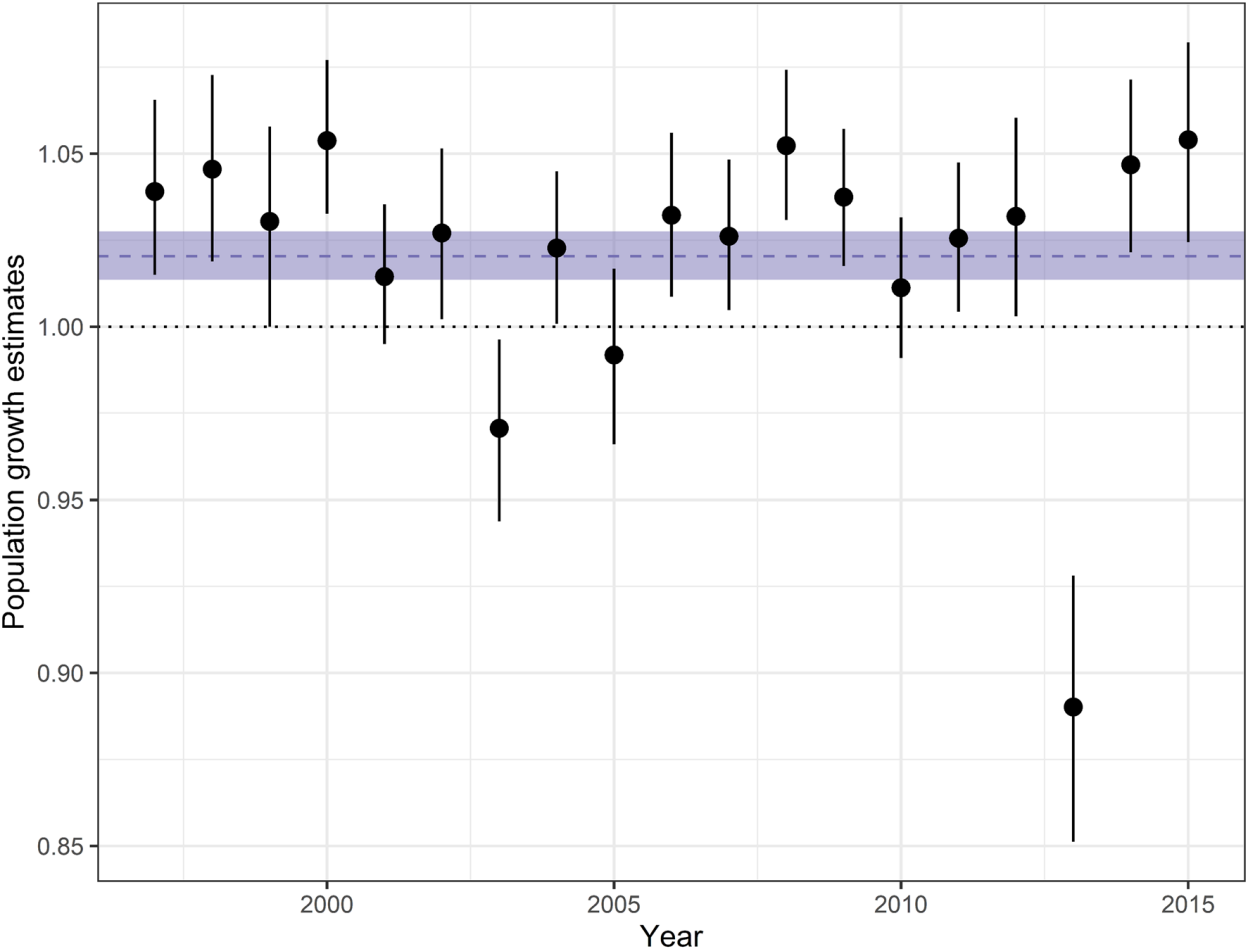
Population growth rate estimates (medians and 95% credible intervals) from IPM, 1997–2015. Population stability (growth rate = 1) is indicated with a dotted line. Average population growth rate (geometric mean across years) is indicated with a dashed line; ribbon indicates 95% CRI.

**Figure 5:**
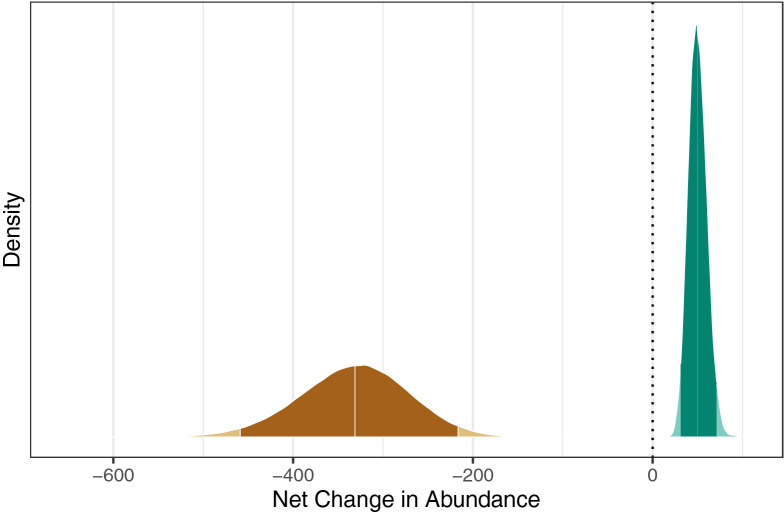
Density plots comparing estimated effect of the year 2013 on abundance (brown) with the net change in abundance in an average year (green). The darker-shaded regions indicate the 95% CRI and the middle vertical break lines within them indicate the median. The dotted line indicates a hypothetical change in abundance of 0 manatees.

Posterior abundance estimates from the IPM ranged from 2014 (in 1997; 95% CRI 1861–2229) to 3019 (2013; 2668–3431) and in general showed a pattern of slow but variable population growth (Fig. 3). Uncertainty in the posterior abundance estimates was considerably smaller than that in the prior abundance estimates for 2011 and 2016 (from the survey analysis, without use of the IPM). The apparent dramatic increase in abundance between 2011 and 2016 based on the abundance survey was tempered by consideration of other information, and the difference in those posterior distributions suggests a smoother pattern of population change. All abundance estimates were considerably higher than the aerial synoptic (meaning general view of the whole) survey counts from the same years. Realized population growth rate estimates varied from 0.89 (2013; 0.85–0.93) to 1.05 (2000; 1.03–1.08) with a geometric mean of 1.02 (1.01–1.03; Fig. 4). Point estimates of realized population growth rates were below 1 in only 3 years (2003, 2005, and 2013); in more than half the years the lower credible limit was also above 1. We estimated that the population declined in 2013 by 331 animals (217–459) (Fig. 5). By contrast, in an average year the population increased by an estimated 50 manatees (31–72).

**Figure 6:**
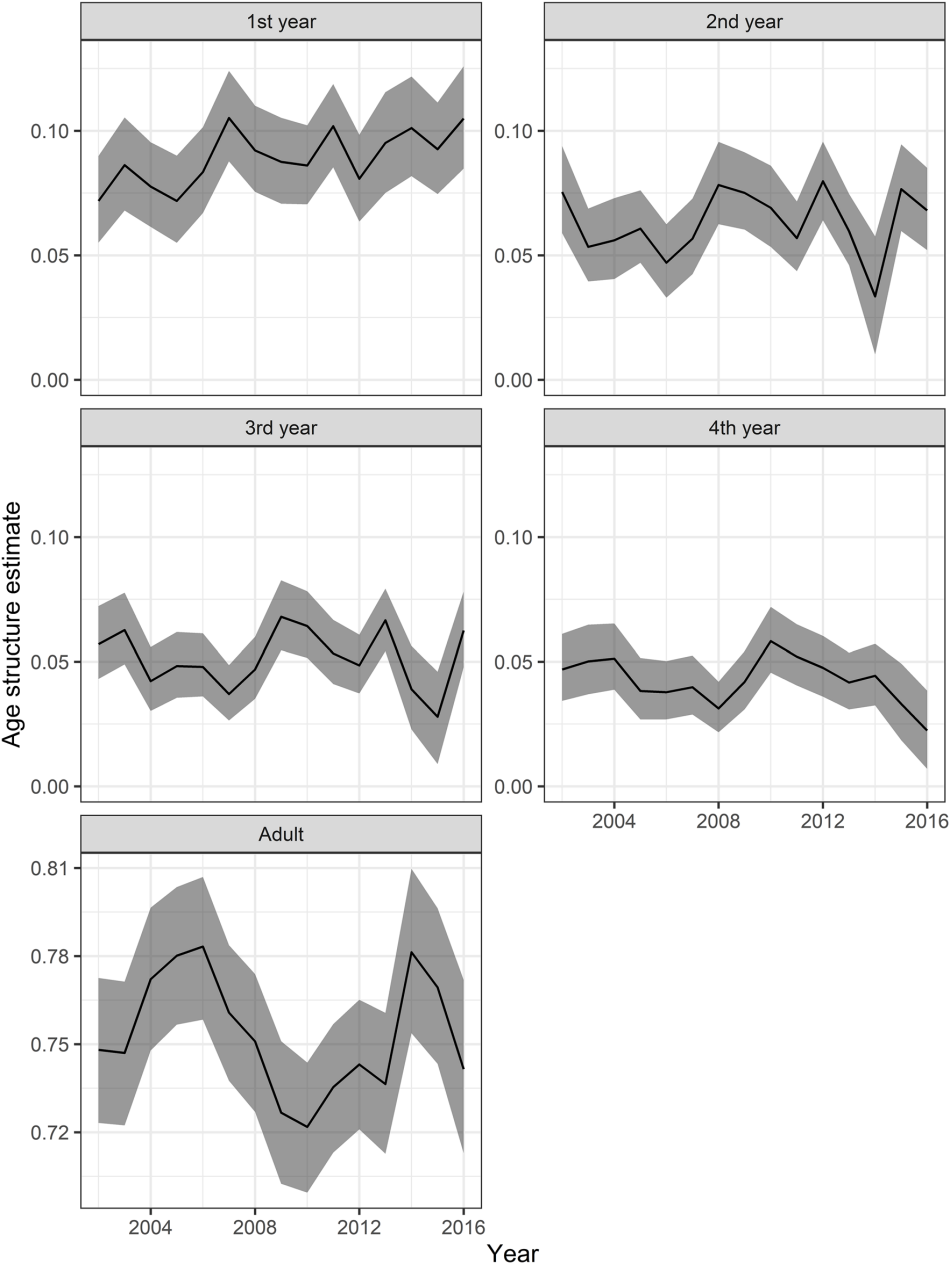
Age class structure estimates from IPM. Black lines indicate median estimates; gray ribbons indicate 95% CRI. Note that the y-axis is scaled differently for adults.

The simulation-based hindcast models also provided estimates of abundance, but with less precision than the IPM (Supplementary Note and Supplementary Figs. S1 and S2, online). The estimates of the number of dead manatees by coarse stage and year from the hindcast models were often inconsistent with the carcass recovery data (Supplementary Fig. S3, online). Estimated age class structure varied over time, but the proportion of adults in the population was always more than 65% (Fig. 6). Cohorts can be seen progressing through the other stages over time; for example, a peak in relative abundance of first-year calves in 2007 translates into a peak in second-year calves in 2008, third-year subadults in 2009, and fourth-year subadults in 2010.

**Figure 7:**
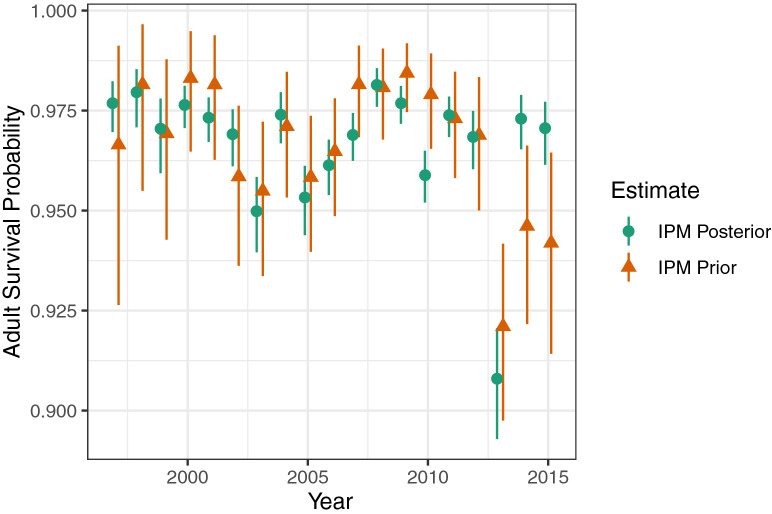
Estimated adult survival probabilities by year. The estimated IPM prior is from the photo-identification analysis of manatees in southwest Florida^11, 52^. The estimated IPM posterior integrates those estimates with carcass recoveries and other data. Symbols indicate medians; line segments indicate 95% credible intervals.

**Figure 8:**
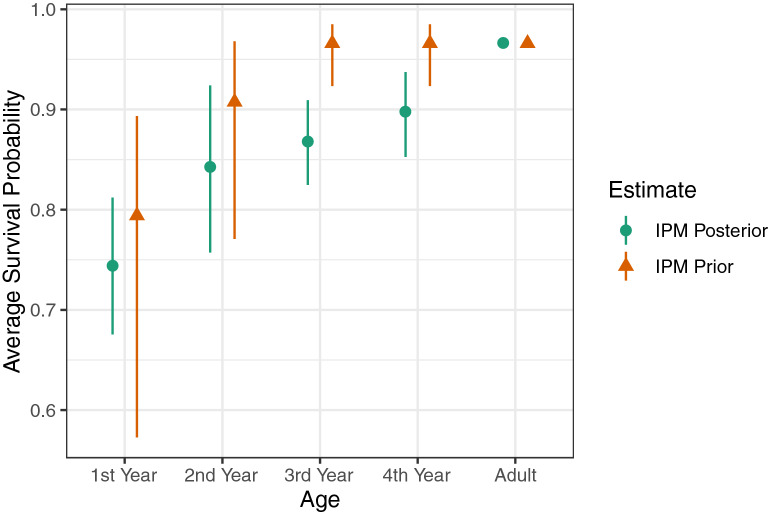
Estimated survival probabilities (averaged across years) by age class. The estimated IPM prior is from the photo-identification analysis of adult manatees in southwest Florida and mortality ratios from a photo-identification analysis of manatees in the Upper St. Johns River^26^. The estimated IPM posterior integrates those estimates with carcass recoveries and other data. Symbols indicate medians; line segments indicate 95% credible intervals.

Posterior estimates of adult survival probability were similar in most years to prior estimates (Fig. 7) but more precise. The last 2 years of the analysis (2014 and 2015) stand out as having low estimates of survival in the prior but close to average estimates of survival in the posterior. Posterior estimates of average calf and subadult survival probability were less than prior estimates and showed a pattern of increase with age (Fig. 8). Except for first-year calves, however, the posterior estimates were not noticeably more precise than prior estimates. Posterior estimates of adult female reproductive probability were mostly very similar to the prior estimates, with little gain in precision (Supplementary Fig. S4, online). The low posterior estimate of reproductive probability for fourth-year subadults (0.00083; 0.000–0.108) was similar to the prior estimate (0.00100; 0.000–0.285).

Estimated average carcass recovery rates were high for subadults (0.948; 0.870–0.982) and adults (0.971; 0.899–0.995) but lower for calves (0.670; 0.477–0.844; Supplementary Fig. S5, online). Estimated recovery rates also varied considerably by year (Supplementary Fig. S6, online). They were lowest in 2013 (calves: 0.293; 0.208–0.415; subadults: 0.784; 0.639–0.896; adults: 0.868; 0.688–0.974) and highest in 2010 (calves: 0.920; 0.740–0.996; subadults: 0.990; 0.958–0.999; adults: 0.995; 0.971–1.000).

Sensitivity analysis for different starting (1997) stage structures showed considerable variation in stage structures for subsequent years (Supplementary Fig. S7, online) and in age class structure for 1997–2001 but little variation in total abundance for any year or in age class structure for 2002–2016 (Supplementary Figs. S8 and S9, online). Sensitivity analysis for different starting abundances showed little variation in total abundance for any year and no variation in stage structure or age class structure (Supplementary Figs. S10–S12, online).

## Discussion

In this study we were able to obtain estimates of parameters that had been missing for the southwest subpopulation, including survival probabilities of younger stages of manatees, recovery rates of manatee carcasses, and abundance in years before and between abundance surveys.

Survival probabilities of younger animals are key parameters in population viability analyses of Florida manatees^11,23,25^. But these probabilities have long been extrapolated from one study of manatees in a small management unit on Florida’s east coast^26^. The average probabilities of juvenile survival estimated here are lower than those obtained from that extrapolation (Fig. 8). Independent estimates of the younger manatee survival probabilities for the southwest management unit will soon be available from genetic mark–recapture–recovery modeling, but such data are not forthcoming for the other three Florida manatee management units, making the approach used here for estimating these probabilities more readily applicable.

In addition, our model provided estimates of the effects of red tide and cold events on the population. The red tide event of 2013, during which 353 carcasses were recovered in the southwest (of which at least 268 were killed by red tide), contributed to an estimated net drop in the population of 331 (217–459) manatees (Fig. 5) for an annual population growth rate of 0.89 (0.85–0.93; Fig. 4). Our results support the finding that such red tide events (classified as intense) affect calves particularly (Supplementary Fig. S13, online)^11^. In contrast, the cold event of 2010, which led to 247 recovered carcasses in the southwest region, did not appear to lead to a net drop in population, according to our model. This may be in part because our prior estimate of adult survival that year was relatively high (Fig. 7), and the model assumes (and estimates) a fixed ratio between age-class survival rates across years (Supplementary Table S1, online). These new estimates can be helpful in communicating the impact these disturbance events had on the population. Unusual mortality events that lead to high carcass counts often attract a lot of attention from the press and the public. The IPM provides a way to put such mortality events in perspective and to answer questions such as “What was the impact of a particular mortality event on the population?” In addition, the average population growth rate (1.02, 1.01–1.03) estimated from our data supports the hypothesis that the manatee population was increasing from 1997 to 2016 (Figs. 3 and 4). This is the first rigorous estimate of historical (realized) population growth rate for this population. This information is complementary to and consistent with the projected population growth rate obtained from the CBM projections^11^.

Our model also provided more precise estimates of many parameters estimated earlier, such as adult survival and abundance for years in which abundance surveys were carried out (Figs. 3 and 7). In some cases, our approach may reduce bias, although it is also possible for IPMs to introduce or increase bias^27^. Possible biases in some input estimates to our model, such as abundance^28,29^ and end-of-time-series survival probabilities^30^, have been noted^28–30^. In some cases, the median estimates obtained from the IPM were substantially different from the original estimates (compare prior abundance survey and posterior estimates in Figs. 3 and 7). The IPM might correct for biases in abundance and end-of-time-series survival estimates, although this idea needs to be further evaluated. Because it includes a recovery model for carcass data, the IPM does not hindcast impossible numbers of deaths, unlike the simulation-based hindcast model (Supplementary Fig. S3, online). The IPM results suggest that these results from the simulation-based hindcast model were off both because the 2011 abundance estimate input was too low and because the survival estimate inputs for juveniles (*s*_1_–*s*_4_) were too high. By integrating multiple sources of information, we are synthesizing the best available information but also hedging our bets by not relying on just one source of data in estimating critical demographic parameters.

Many of our posterior estimates are consistent with other published results for Florida manatees. Our estimates of realized population growth rates (Fig. 4) are similar to the projected population growth estimates from the CBM and consistent with general trends of growth in synoptic and carcass counts. Our estimates of age structure (Fig. 6), although variable over time, are consistent with the asymptotic stable age structure that projecting from a simple matrix model would provide. Our estimates of the mortality effects of the 2013 red tide (Supplementary Fig. S13, online) are similar to those from the CBM. The pattern of our estimated recovery probabilities by coarse stage (Supplementary Fig. S5, online) is consistent with an earlier estimate of age-specific recovery rates relative to (unknown) adult recovery probability^31^, although our estimates of subadult and adult recovery probabilities are closer to 1 than we expected. The high estimates of recovery probability may be due to the IPM attempting to harmonize partially incompatible model components (Supplementary Fig. S3, online). When model components generate incompatible results, either due to model misspecification or not referencing exactly the same populations, an IPM must reconcile those results. This reconciliation can generate bias in some estimates, although the generally higher precision of IPM estimates may still mean higher accuracy. Ground truthing or other research may be needed to determine whether FWC is actually recovering such a high proportion of manatee carcasses.

The results of this study are relevant to the management of Florida manatee populations. The manatee recovery plan used by the USFWS under the Endangered Species Act relies on several metrics that can be obtained from the IPM, such as realized population growth rates and population size. The IPM provides one of the most rigorous assessments to date for these quantities and may be used by natural resource managers in assessing the status of the manatee population. It can also be used to update key model parameters of the CBM, which at present is the primary population assessment tool for managers.

Another important regulatory framework relevant to marine mammal conservation in the United States is the Marine Mammal Protection Act. Here again, an IPM can help in addressing some of the act’s requirements. Indeed, the act specifies a formula for computing potential biological removal (*PBR*; the maximum number of animals that can be removed from a stock while allowing it to reach or remain at its optimum sustainable population)^32–35^

1 $$\begin{aligned} PBR & = N_{min} \frac{{R_{max} }}{2}F_{r} \\ N_{min} & = \frac{{\hat{N}}}{{\exp \left( {0.842\sqrt {\log \left( {1 + {\text{CV}} \left( {\hat{N}} \right)^{2} } \right)} } \right)}} \\ \end{aligned}$$

where *N*_*min*_ is the minimum population abundance estimate (20th percentile of abundance estimate distribution), *R*_*max*_ is the theoretical maximum rate of increase for the stock, *F*_*r*_ is a recovery factor (generally 0.5 for threatened species, but see Moore et al.^35^), and $\hat{N}$ is the point estimate of population abundance. Based on our estimate from the last year of the analysis (2016), *N*_*min*_ for the southwest population of Florida manatees is about 2780. This is lower than *N*_*min*_ would be based on the abundance survey (prior) estimate from the same year (about 3140); $CV\left(\hat{N}\right)$ from the IPM posterior was lower than from the prior (Supplementary Fig. S2, online) but $\hat{N}$ was as well (Fig. 3). Estimation of *R*_*max*_ requires extrapolating growth rates to conditions of low population density and absence of anthropogenic mortality; our IPM is not designed for that purpose, but future extensions could be developed to address this need. A merging of our IPM, or other matrix model approach, with an allometric approach to estimating *R*_*max*_ would allow a more accurate estimate of this parameter^36^. Both matrix model (individual population) and allometric (cross population) approaches to estimating *R*_*max*_ are strongly affected by biases caused by using empirical estimates of adult survival instead of what adult survival would be under ideal conditions; however, these biases run in opposite directions, so an integration of these approaches greatly reduces any bias in *R*_*max*_^36^.

Another benefit of the IPM is its usefulness for planning monitoring activities, including how to allocate resources to various aspects of the monitoring program, such as aerial surveys, photo-identification, genetic sampling, and carcass recovery. Various sampling scenarios (e.g., 40% of carcasses recovered; 200 genetic samples per year; one aerial survey every 5 years) can be combined with simulated data generated under those scenarios to see how the accuracy of model parameter estimates differs among scenarios. Trade-offs between parameter accuracy/precision and budget allocation can then be examined to improve monitoring efficiency. Optimizing the sampling with an IPM also makes sense in the context of targeted monitoring for adaptive management^37^. In such applications, the IPM can be used to estimate state variables (e.g., abundance) that keep track of system changes, allow managers to implement state-dependent decisions, and update beliefs about which model is the best approximation of reality (through Bayes theorem)^37,38^. A now classic example of an implementation of this adaptive management process is for the sustainable harvesting of waterfowl in North America^37^, where the optimal state-dependent harvest policies are driven, at least partially, by waterfowl abundance. IPMs are now being used to increase precision of abundance and other state variables in adaptive management of waterfowl^39,40^.

A monitoring component that could be streamlined is the carcass-recovery and necropsy program. The present protocol is that almost all carcasses reported must be recovered and necropsied, which, along with the growth in the manatee population, is making this program increasingly labor-intensive and expensive. The IPM gives us the first true estimates of carcass recovery probabilities for Florida manatees. These estimates are now being used by FWC in evaluating and improving the efficiency of these programs.

Monitoring populations of marine mammals involves special challenges, such as the difficulty, cost, and risk to researchers involved in counting the population, often through aerial surveys. Several other studies that involved the development of IPM for marine mammals^16,41–43^ had at least one thing in common with ours: population surveys were not conducted every year, which differs from most IPMs used for terrestrial birds and mammals. Our approach, like those applied to other marine mammals, could be valuable for filling in abundance estimates for other sirenians and small cetaceans, where estimating survival and reproductive probabilities from mark–recapture data is often easier than obtaining abundance estimates. As explained earlier, the IPM can then be used to determine the optimal frequency of surveys and optimal spatial sampling effort (e.g., how much area to survey and how many survey visits at each location to estimate detection)^28^.

Studies of other marine mammals^16,41–43^ collected explicit data on age or stage structure, while for manatees, reliable data were not available for these parameters. We were able to estimate age class structure for the years 2002–2016 using neither stage structure data nor particularly informed priors (Fig. 6). This is likely because of the weak ergodic theorem of demography, which shows that the initial stage structure becomes less relevant with more years of known (or, in our case, estimated) survival and reproductive probabilities^3,44^. Our approach may be useful for other marine species without reliable stage structure information. Modeling stage structure and transient dynamics can be important to improving understanding of the dynamics of wild populations and can have important management implications. For instance, Johnson et al.^45^ found that the initial stage structure could have substantial policy consequences for the management of an invasive species.

Our IPM and the associated input models are based on a series of assumptions (Supplementary Table S1, online). One of the assumptions of the IPM is the independence of the data sources for the input analyses. This assumption is violated in our case; the adult survival analysis shares carcass data with the recovery analysis and mark–recapture data with the reproductive analysis. Two simulation studies^17,46^ found that violating this assumption had little effect, but as their analyses were not identical to ours, this assumption violation still might diminish the accuracy of our estimates. Simulations by Rieke et al.^47^ show that assumption violations in one of the model components can dramatically reduce the accuracy of estimates of latent parameters. Therefore, in our case, the estimates of juvenile survival, recovery probabilities, and abundance in years without abundance surveys should all be interpreted cautiously.

There are several possible extensions of this model, for example for use in the other three Florida manatee management units (Fig. 1). Because we are uncertain about winter within-coast manatee distribution^29^, two coast-wide IPMs that each jointly model the two management units on that coast might be most appropriate. With an initial abundance distribution and yearly vital rate estimates for each management unit (possibly including movement rates between regions, if they become available), subsequent coast-wide abundance estimates could be shared between them. This would allow relaxation of the assumption that the proportion of the winter population in each of the two management units remains fixed over time.

Possible extensions could demonstrate whether and to what extent the IPM decreases bias in input estimates, through simulating estimates with known biases and carcass data, running the IPM with the simulated data, and repeating this process many times. One could similarly test the model’s robustness to different assumption violations.

Preliminary analyses suggest that our use of earlier analyses as priors in the integrated model does not bias results but that it might reduce precision. Therefore, it may be useful to estimate more parameters from data directly within a future version of this IPM. In addition, incorporating additional data sources (such as genetic mark–recapture and age estimates using tympanoperiotic ear bones) could improve parameter estimation. Since each parameter can have only one prior, this too requires performing more of the data analysis within the IPM.

Despite these limitations, we believe that this manatee IPM is the most rigorous means of retrospective assessment of the population dynamics of the Florida manatee. Because the model is modular (e.g., abundance module, survival module), as each module is improved, the model as a whole is improved. This offers a compelling framework within which to synthesize and update information about population dynamics. We have shown here that an IPM can be used: (1) to infer historical trends in abundance, improving our understanding of population dynamics and therefore our ability to forecast; (2) to model the transient dynamics of stage distribution, which can be important to some populations; (3) to assess the conservation status of wild populations and to communicate that information to stakeholders (e.g., we can now quantify the impact of the 2013 red tide event on the manatee population); and (4) to improve allocation of effort in complex monitoring programs.

Our modeling frameworks are relevant to population status assessment protocols for management and conservation, such as recovery plans under the Endangered Species Act and potential biological removal under the Marine Mammal Protection Act. Other marine mammal conservation programs, such as that of the Hawaiian monk seal, also have complex monitoring components^48^. We hope that our ideas can inform other programs that focus on the conservation of marine mammals.

## Methods

### Study population

The Florida manatee is a highly mobile, long-lived marine mammal. Its population is divided into four management units or regions^49^, based on where individuals aggregate in the winter: northwest, southwest, Atlantic coast, and Upper St. Johns River (Fig. 1). We focus here on the southwest management unit.

### Population model

For the retrospective analyses we used a modified version of the basic model of the CBM developed by Runge et al.^11,23^. We considered a population with 10 stages, assuming a December census averaging about half a year after birth, labeled for the subsequent year. Like Runge et al. we used a two-sex model (Fig. 2). This population model can be expressed as a series of stochastic binomial draws and deterministic difference equations:

2 $$\begin{aligned} & N_{f2} \left[ {t + 1} \right]\sim {\text{Bin}} \left( {S_{1} \left[ t \right],0.5} \right) \\ & N_{f3} \left[ {t + 1} \right]\sim {\text{Bin}} \left( {N_{f2} \left[ t \right] ,s_{2} \left[ t \right]} \right) \\ & N_{f4} \left[ {t + 1} \right]\sim {\text{Bin}} \left( {N_{f3} \left[ t \right] ,s_{3} \left[ t \right]} \right) \\ & N_{p} \left[ {t + 1} \right] = \left( {S_{f4} \left[ t \right] - G_{4} \left[ t \right]} \right) + \left( {S_{p} \left[ t \right] - G_{p} \left[ t \right]} \right) \\ & N_{c} \left[ {t + 1} \right] = G_{4} \left[ t \right] + G_{p} \left[ t \right] + G_{b} \left[ t \right] \\ & N_{b} \left[ {t + 1} \right] = S_{c} \left[ t \right] + \left( {S_{b} \left[ t \right] - G_{b} \left[ t \right]} \right) \\ & N_{m2} \left[ {t + 1} \right] = S_{1} \left[ t \right]\; - N_{f2} \left[ {t + 1} \right] \\ & N_{m3} \left[ {t + 1} \right]\sim {\text{Bin}} \left( {N_{m2} \left[ t \right] ,s_{2} \left[ t \right]} \right) \\ & N_{m4} \left[ {t + 1} \right]\sim {\text{Bin}} \left( {N_{m3} \left[ t \right] ,s_{3} \left[ t \right]} \right) \\ & N_{ma} \left[ {t + 1} \right] = S_{m4} \left[ t \right] + S_{ma} \left[ t \right] \\ \end{aligned}$$

where *N* corresponds to the number of manatees in a stage; *s* indicates survival probability; subscripts *f* and *m* indicate sex; and subscripts 1, 2, 3, 4, *p*, *c*, *b*, and *a* denote first, second, third, fourth, prebreeder, mothers-with-calf, breeder, and adult stage classes, respectively. *S* and *G* correspond to results of binomial draws, numbers of manatees in a stage that survived or bred, respectively, in a time step, tracked to model the demographic stochasticity processes of survival and reproduction:

3 $$\begin{aligned} & S_{1} \left[ t \right]\sim {\text{Bin}} \left( {N_{c} \left[ t \right] ,s_{1} \left[ t \right]} \right) \\ & S_{f4} \left[ t \right]\sim {\text{Bin}} \left( {N_{f4} \left[ t \right] ,s_{4} \left[ t \right]} \right) \\ & S_{p} \left[ t \right]\sim {\text{Bin}} \left( {N_{p} \left[ t \right] ,s_{p} \left[ t \right]} \right) \\ & S_{c} \left[ t \right]\sim {\text{Bin}} \left( {N_{c} \left[ t \right] ,s_{a} \left[ t \right]} \right) \\ & S_{b} \left[ t \right]\sim {\text{Bin}} \left( {N_{b} \left[ t \right] ,s_{a} \left[ t \right]} \right) \\ & S_{m4} \left[ t \right]\sim {\text{Bin}} \left( {N_{m4} \left[ t \right] ,s_{4} \left[ t \right]} \right) \\ & S_{ma} \left[ t \right]\sim {\text{Bin}} \left( {N_{ma} \left[ t \right] ,s_{a} \left[ t \right]} \right) \\ & G_{4} \left[ t \right]\sim {\text{Bin}} \left( {S_{f4} \left[ t \right] ,\gamma_{4} \left[ t \right]} \right) \\ & G_{p} \left[ t \right]\sim {\text{Bin}} \left( {S_{p} \left[ t \right] ,\gamma_{p} \left[ t \right]} \right) \\ & G_{b} \left[ t \right]\sim {\text{Bin}} \left( {S_{b} \left[ t \right] ,\gamma_{b} \left[ t \right]} \right) \\ \end{aligned}$$

where *γ* represents reproductive probability. Because first-year calves are not tracked separately in the model but are included in the mothers-with-calf stage, the total abundance in year *t* can be calculated as:

4 $$\begin{aligned} N_{tot} \left[ t \right] & = N_{f2} \left[ t \right] + N_{f3} \left[ t \right] + N_{f4} \left[ t \right] + N_{p} \left[ t \right] + 2N_{c} \left[ t \right] + N_{b} \left[ t \right] \\ & \quad + \,N_{m2} \left[ t \right] + N_{m3} \left[ t \right] + N_{m4} \left[ t \right] + N_{ma} \left[ t \right] \\ \end{aligned}$$

We use the term *stage* to refer to one of the 10 stages in Eq. (4). We use the term *coarse stage* to refer to calves (first- and second-year manatees), subadults (third- and fourth-year manatees), or adults (all other stages), without regard to sex. We use the term *age class* to divide manatees into first-year, second-year, third-year, fourth-year, and adult stages, without regard to sex.

We developed several simple simulation-based hindcast models as our initial approach to applying this population model retrospectively (Supplementary Methods online).

### Input data and estimates

Before 2011, the only statewide manatee counts were done using aerial synoptic surveys, carried out most years during the coldest part of winter when manatees tend to aggregate at warm-water sites^28,50,51^, starting in 1991. The synoptic surveys are each flown over a short time (one to a few days), generally with a single observer per airplane. Although these surveys are intended to be comprehensive, they do not account for the number of manatees that are missed because they are absent from surveyed sites, present but underwater or otherwise not available to be detected by the observer, or available yet not detected by the observer. In the retrospective analyses, we used synoptic survey results from southwest Florida as counts that represent the lower bounds for abundance.

In 2011, a new aerial survey method was implemented that accounts for manatee presence at survey locations, availability, and detection, using a stratified random plot design, independent estimates of manatee availability, and a double observer protocol, respectively^28^. The population abundance estimate for southwest Florida in 2011 was obtained from Hostetler et al.^29^, which was an update of the estimate made by Martin et al.^28^ We approximated this estimate and its uncertainty using a lognormal distribution, with mean 7.72 and SD 0.166 on the log scale. In estimating the initial 2016 abundance (another survey was conducted in early December 2015), Hostetler et al. questioned whether the estimated distribution of manatees between southwest and northwest Florida in early December 2015 was representative of the midwinter distribution and concluded it likely was not^29^. Therefore, instead of using the estimate for southwest Florida for December 2015, we multiplied the estimate for the entire west coast of Florida by an estimated proportion of those animals in the southwest, obtained from the synoptic surveys, including uncertainty reflected as temporal variability in synoptic proportions. We approximated this product and its uncertainty with a lognormal distribution with mean 8.16 and SD 0.132 on the log scale.

Annual adult survival probability estimates were obtained from the Barker robust design mark–recapture model^52^. The data consisted of photo-documented sightings of live marked individuals under a traditional sampling framework at primary sampling locations during winter (Fig. 1), and identification of recovered carcasses. Substantial temporary emigration of individuals from study areas can bias estimates of survival rates for long-lived species, particularly at the end of the time series. The Barker robust design models these types of emigration, reducing bias and increasing precision^30^.

Annual estimates of reproductive probability were obtained using a multistate robust design mark-recapture model^53^. The data for this model were photo-documented sightings of marked adult females and associated data indicative of calving. The reproductive state of individual female manatees can be uncertain even when the female is detected, resulting in biased estimates if one assumes they did not calf that year. This multistate model adjusts for reproductive state using hidden Markov processes, both increasing precision and reducing bias^54,55^. We used annual estimates from 1997 to 2015 for both survival and reproductive probabilities, using year as a random effect (method-of-moments variance component) on the sin-link coefficients^56^. Maximum likelihood shrinkage estimates, estimates of variance, and sampling covariances for these coefficients were obtained from program MARK.

The only direct estimates of Florida manatee survival rates for calves and subadults (*s*_1_–*s*_4_) come from the Upper St. Johns River region and the years 1979/80–2000/01^26^. That study found no evidence of a difference between survival rates of subadults (*s*_3_ and *s*_4_) and adults (*s*_*p*_ and *s*_*a*_), but lower survival for each of the calf stages (*s*_1_ and *s*_2_). Following the CBM^11^, we constructed mortality ratios between adults and other age classes within the IPM, using as prior distributions the survival estimates (with associated uncertainty) of that analysis. The IPM assumes that those mortality ratios are the same in all years.

We used the estimate of the probability of reproduction for fourth-year females (*γ*_4_) from Runge et al.^23^ The CBM assumes no temporal variation in *γ*_4_; we maintained that assumption. We diverged from the CBM, which assumes that *γ*_*p*_ does not vary over time and is slightly less than the average *γ*_*a*_[*t*], by assuming that *γ*_*p*_[*t*] = *γ*_*a*_[*t*].

Carcass counts for southwest Florida in each year were obtained from FWC’s manatee mortality database (http://myfwc.com/research/manatee/rescue-mortality-response/mortality-statistics/). Carcasses were divided into coarse stage by carcass length (calves: 151–235 cm; subadults: 236–265 cm; and adults: 266 cm or more). Carcasses less than 151 cm in length (*n* = 1391) were defined as perinatal and were excluded from the analysis. Carcasses of unknown length (*n* = 79; mostly too decomposed or verified but not recovered) were also excluded from the analysis.

### IPM

The general methods of integrated population modeling have been well described^13,14^. Our IPM differs from most others in that we use previous estimates of abundance, adult survival probabilities, and female reproductive probabilities as prior distributions, an accepted alternative to directly integrating those analyses into the model^57–59^.

To provide a prior probability distribution on initial (1997) abundance, we ran several simulation-based hindcast models, some working backward from the 2011 abundance estimate and others from the 2016 abundance estimate (Supplementary Methods, online). We combined model runs across models and fit a lognormal distribution to all the 1997 abundances generated. Preliminary testing suggested that doubling the SD of the log from this estimate provided a better measure of our true uncertainty about initial abundance: mean 7.38 and SD 0.49 on the log scale.

In 1996 the manatee population in southwest Florida suffered at least two stochastic shocks: a cold winter and a red tide event classified as intense^11,60^. For this reason, we did not think that the stable stage distribution would necessarily be a good approximation of the true stage distribution in 1997. The latest CBM report provides estimates of the effects of these types of events on manatee mortality, by coarse stage^11^. We used these estimates to shift the initial stage distribution from a stable distribution in one of the simulation-based hindcast models (Supplementary Methods, online). We used the estimates of 1997 stage structure from that model, modeled with a Dirichlet distribution, and with variance quadrupled to allow for additional uncertainty (the population may not have reached the stable distribution at the beginning of 1996 either). We tested the sensitivity of abundance, stage structure, and age structure to uncertainty in the initial abundance and stage structure (Supplementary Methods, online).

A type of data we directly modeled in the IPM was recovered carcasses. We used a binomial model:

5 $$C_{s} \left[ t \right]\sim {\text{Bin}} \left( {M_{s} \left[ t \right],r_{s} \left[ t \right]} \right)$$

where *C*_*s*_[*t*] is the number of carcasses recovered in year *t* of coarse stage *s*, *M*_*s*_[*t*] is the number dead in that year and stage, and *r*_*s*_[*t*] is the associated recovery probability. We tested several models for *r* and used binary inclusion factors^61,62^ and parameter credible intervals to settle on additive effects of coarse stage and year, with year as a random effect.

### Implementation

We implemented the IPM using the R (version 3.5.1) package NIMBLE (version 0.8.0), an MCMC implementation using the BUGS language^63,64^. We ran each version of the model for three chains of 6 million iterations after a burn-in of 2 million each and thinned the results by 50. We tested for convergence using Gelman-Rubin statistic and visual examination of the chains^65^. All models presented converged successfully.

To minimize mean absolute deviation, we used the medians of the posterior distributions as the point estimates for all parameters (except for binary inclusion factors, for which we used means)^66^. We present the 0.025 and 0.975 quantiles of the posterior distributions as the 95% Bayesian credible intervals for each parameter.

We used this IPM to estimate several parameters: total year-specific abundance for 1997–2016; realized year-specific population growth rates for 1997–2015 ($\lambda \left[t\right]=\frac{{N}_{tot}\left[t+1\right]}{{N}_{tot}\left[t\right]}$); the geometric mean population growth rate; the net population change in an average year and in 2013; annual year-specific age class structure for 2002–2016; age-class-specific and, in many cases, year-specific survival and reproductive probabilities for 1997–2015; and coarse stage–specific and year-specific carcass recovery probabilities for 1997–2015.

## Supplementary Information

Supplementary Information

## Data Availability

Code and data for the IPM and hindcast models are available at: 10.5066/P98835OJ.
