# Supplementary material for: Reconstructing population dynamics of a threatened marine mammal using multiple data sets
Source: Sci Rep. 2021 Jan 29;11:2702. doi: 10.1038/s41598-021-81478-z (PMC7846604; doi:10.1038/s41598-021-81478-z)
Supplement: Supplementary file 1 — Supplementary Information [file 41598_2021_81478_MOESM1_ESM.pdf]

-Supplementary Information

Reconstructing population dynamics of a threatened marine mammal using multiple data sets

Jeffrey A. Hostetler[1,2*], Julien Martin[3,4], Michael Kosempa[1], Holly H. Edwards[1], Kari Rood[1], Sheri L. Barton[5] & Michael C. Runge[6]

[1]Fish and Wildlife Research Institute, Florida Fish and Wildlife Conservation Commission, St. Petersburg, FL 33701, USA

[2]Current: Patuxent Wildlife Research Center, U.S. Fish and Wildlife Service, Laurel, MD 20708, USA

[3]Wetland and Aquatic Research Center, U.S. Geological Survey, Gainesville, FL 32653, USA

[4]U.S. Geological Survey, St. Petersburg Coastal and Marine Science Center, St. Petersburg, FL 33701, USA

[5]Mote Marine Laboratory, 1600 Ken Thompson Parkway, Sarasota, FL 34236, USA

[6]Patuxent Wildlife Research Center, U.S. Geological Survey, 12100 Beech Forest Road, Laurel, MD 20708, USA

[*]email: Jeffrey_Hostetler@fws.gov

Supplementary Methods

Simulation-based hindcast model

This approach takes an abundance estimate from one year, and projects the population backward and/or forward using vital rate estimates from each year, incorporating both uncertainty in the abundance estimate and uncertainty (including some covariance) in the vital rates. Because this approach can only use one abundance estimate, we projected separately from each of our two abundance estimates (2011 and 2016).

We accounted for uncertainty associated with the initial abundance estimate by assuming a lognormal distribution. We accounted for sampling covariance in survival probability and in reproductive probability estimates between years using a Cholesky decomposition[1]. We followed Runge et al.[2,3] and discarded any iterations for which γ_4 was greater than the average $\gamma_p[t]$. We also discarded any iterations for which abundance in any year was less than the synoptic count for that year.

In 1996 a population shock resulted from an intense red tide event and a cold winter. Therefore, we did not feel it was necessarily appropriate to start the population at a stable stage structure in 1997. We considered several scenarios for stage structure with which to start the hindcast in 1997: 1) stable stage structure based on the averages of vital rates over the years; 2) stage structure based on an additional mortality rate analysis from carcass data for 1996[3]; 3) using a Dirichlet distribution to choose a random stage structure each iteration; and 4) picking the stable stage structure associated with a random year's vital rates each iteration. Scenarios 2 and 3 are described in more detail below.

The **additional mortality rate analysis** used changes in proportions of carcasses with different causes of death to estimate additional mortality due to red tide and cold; it incorporated uncertainty from recovered carcasses for which cause of death was unknown[3]. Additional cold mortality for Florida manatees from 1996 to 2013 was estimated by winter severity, coarse stage, and warm-water habitat quality, with some additional mortality rates fixed to 0. We used estimates from the same report that 9.3% of manatees in the southwest management unit spend winters in low-quality warmwater habitat, 87.7% in medium-quality warmwater habitat, and 3% in high-quality warmwater habitat. Additional red tide mortality for the southwest region was estimated by red tide intensity and calves vs. subadults and adults. We incorporated uncertainty from the additional mortality analysis by sampling from the posteriors of the additional mortality estimates. Additional mortality rates for calves, subadults, and adults were subtracted from average survival probabilities across years to obtain estimates of 1996 survival probabilities. We assumed that 1996 reproductive probabilities were unaffected by the events and used averages across years. We projected the population from an assumed stable stage distribution in 1996 for one year and divided by the sum to obtain the stage distribution for 1997.

For the **Dirichlet approach** we took the estimated stable stage structure (0.035, 0.030, 0.028, 0.073, 0.092, 0.242, 0.035, 0.030, 0.028, and 0.407 for stages *f*2, *f*3, *p*, *c*, *b*, *m*2, *m*3, *m*4, and *ma*, respectively) and divided by the smallest of them to obtain the parameters for the Dirichlet distribution for stage distribution in 1997.

None of the stage structure approaches are ideal. The Dirichlet approach, for example, almost certainly overstates uncertainty in initial stage structure while the other approaches likely understate it. Yet in sensitivity analysis we found that all scenarios resulted in similar total abundance estimates for all years and similar age class structure estimates after 2001. We present results below using the random year's stable stage structure hindcast from both abundance estimate starting points.

Because the population model is nonlinear, finding hindcast solutions was challenging, especially within the context of statistical uncertainty. Below are the steps for our hindcast method, hindcast from the 2011 abundance estimate and using the random year's stable stage structure approach:

Step 1: Sample from the probability distributions for abundance in 2011 ($Nr2011$) and vital rates for each year a) between 1997 and 2010 ($\mathbf{V}_{1997–2010}$) and b) between 2011 and 2015 ($\mathbf{V}_{2011–2015}$). Choose random year between 1997 and 2015, build matrix model based on that year's sampled vital rates, and calculate stable stage distribution based on that matrix (vector $\mathbf{S}_r$).

Step 2: Use an optimization routine (**R** function optimize[4]) to find the total abundance in 1997 ($Ni1997$), which, when multiplied by $\mathbf{S}_r$ to get abundance for each stage (vector $\mathbf{N}_{1997}$) and then projected forward to 2011 using the vital rates $\mathbf{V}_{1997–2010}$, produces a total 2011 abundance that is the same as $Nr2011$.

Step 3: Project stage-specific abundance from year 1997 ($\mathbf{N}_{1997}$ from Step 2 through year 2016 (with vital rates $\mathbf{V}_{2000–2010}$ and $\mathbf{V}_{2011–2015}$ from Step 1 and the projection model described in the Methods section *Population model*). Compute total abundance for each year. If the total abundance for any year is less than a synoptic count from that year, discard these results and return to Step 1.

Step 4: Repeat Steps 1–3 5,000 times to create a distribution of stage-specific abundances for each year.

Similar steps were used to hindcast from the 2016 abundance estimate.

Because this process keeps track of abundance in each stage class, estimates of stage distributions can be derived for each year as the ratio of the number of animals in stage class s divided by total abundance. Age class distributions can be similarly derived. The number of deaths by stage expected for each year can be estimated as the mortality rate times the abundance for that stage and year. These were summed to obtain the number of deaths expected by coarse stage for each year, which was compared to the carcass counts by coarse stage and year.

Sensitivity Analysis

We ran four additional scenarios on initial (1997) abundance, all based on the same suite of eight model results from the simulation-based hindcasts (see above and main methods). The first of these involved multiplying all input abundance estimates by 0.9 before estimating the lognormal distribution; the second involved multiplying them by 1.1. As these modifications changed the mean on the log scale but not the SD, for the third and fourth scenarios we changed only the SD on the log scale. For the third scenario, we didn't multiply the resulting SD on the log scale by anything (it was multiplied by 2 in the main scenario) but used it directly as the prior. For the fourth scenario, we multiplied it by 3.

We ran four other scenarios on the initial stage distribution, which in the main scenario was based on the additional mortality rate analysis from the simulation-based hindcast models (see above and main methods). The first scenario was based on the stable stage structure hindcast model results, the second on the Dirichlet hindcast model results, and the third on the random year hindcast model results. The fourth scenario combined all four types of hindcast results to compute the 1997 stage distribution and uncertainty. For the first of these scenarios, we quadrupled the variance of the resulting Dirichlet, as we did for the main scenario. For the other three, we judged that the variance was already high enough and left it unchanged. Which abundance estimate the hindcast was from (2011 or 2016) had little effect on the 1997 stage distribution, so we used only the 2016 hindcasts here.

Supplementary Note: Hindcast Projection Results

The abundance estimates from the 2011 and 2016 hindcasts were less precise than the IPM estimates (Supplementary Figs. S1 and S2). The 2016 hindcast abundances for each year were higher than the 2011 hindcast abundances from the same year. As a result, the synoptic count trimming affected the 2011 hindcast but not the 2016 hindcast, making the former more precise in most years. The CV for each hindcast was lowest for the year hindcast from, whereas the IPM minimized its CV in 2000 (Supplementary Fig. S2).

Many estimates of number of deaths from the hindcasts were lower than the number of carcass recoveries from the same year and coarse stage, which is impossible (Supplementary Fig. S3). These inconsistencies happened more often from the 2011 hindcast than from the 2016 hindcast, and more often for subadults than for calves or adults. The IPM resolved these inconsistencies by updating the estimates for vital rates and abundance estimates.

Supplementary Figure S1. Annual abundance estimates, 1997–2016, from IPM (IPM posterior), the two aerial abundance surveys, and the simulation-based hindcast projections starting with each abundance survey estimate (2011 and 2016). Symbols indicate medians and line segments 95% credible intervals. Synoptic survey counts are also shown for reference.

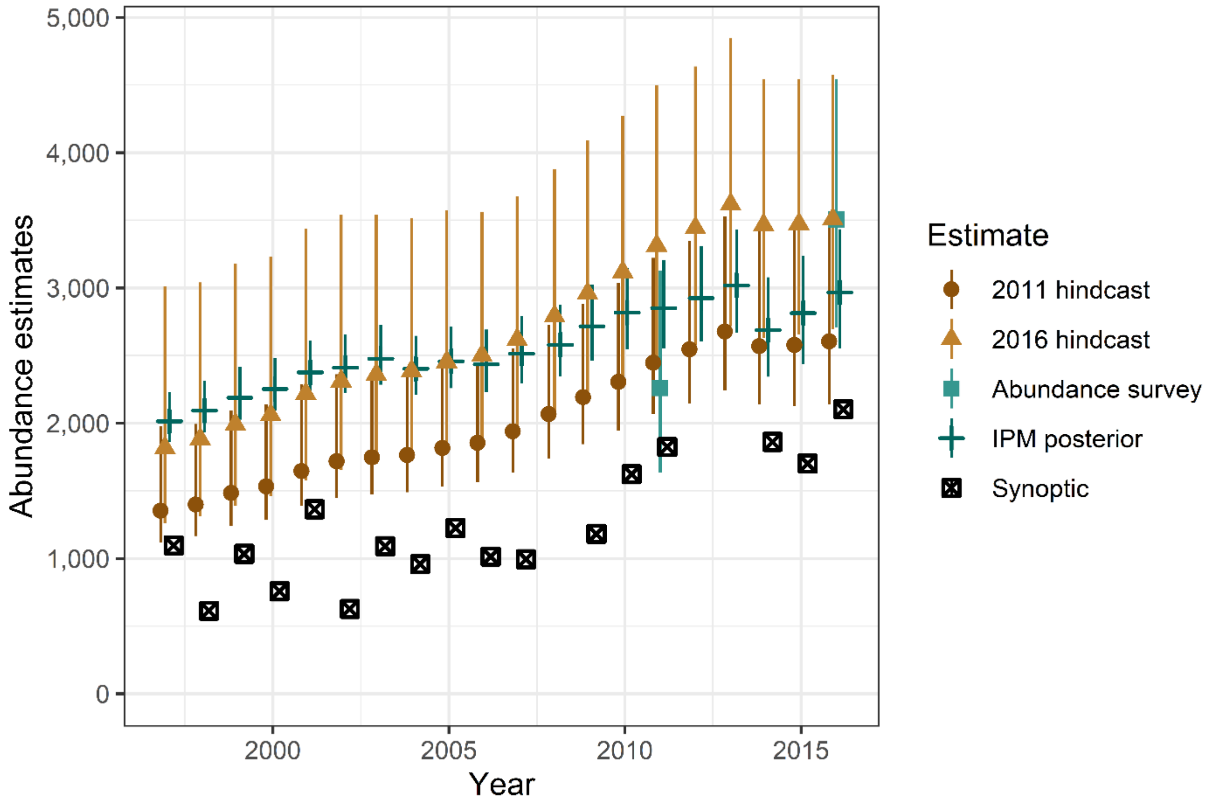

Supplementary Figure S2. Coefficients of variation from annual abundance estimates, 1997–2016, from IPM (IPM posterior), the two aerial abundance surveys, and the simulation-based hindcast projections starting with each abundance survey estimate (2011 and 2016).

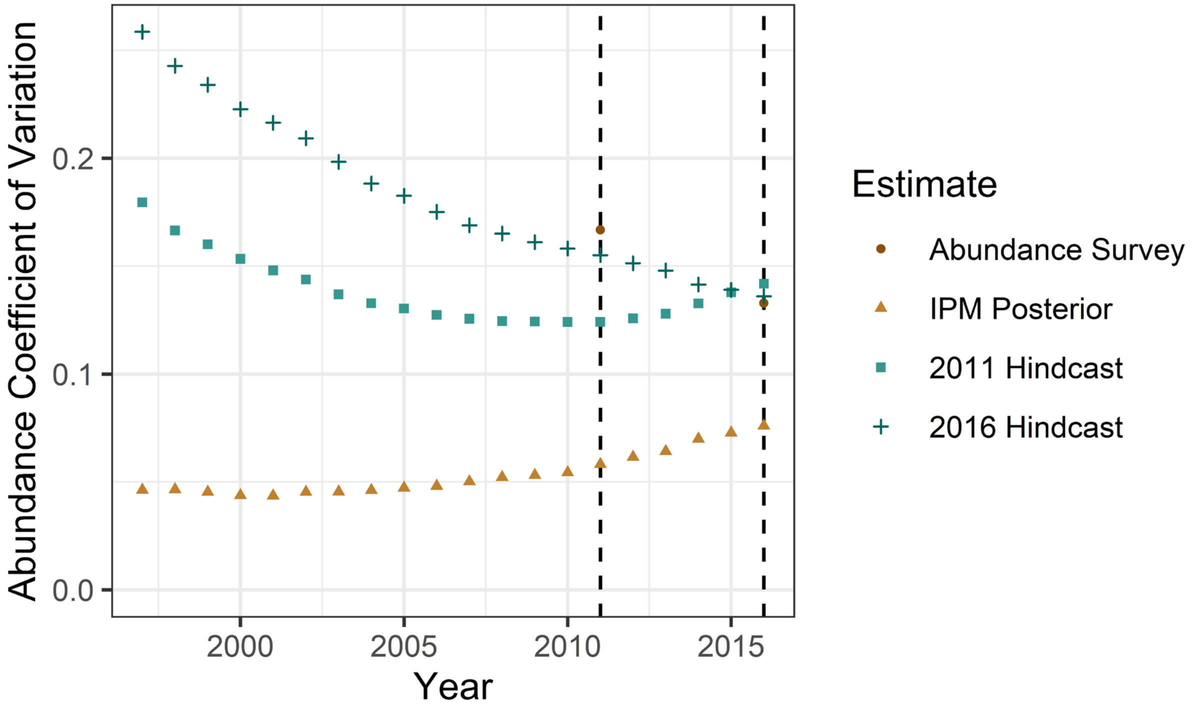

Supplementary Figure S3. Observed recoveries versus expected mortality for the years 1997–2015 by coarse stage for the hindcast projections starting with each abundance survey estimate (2011 and 2016). Dots indicate medians and the line segments 95% credible intervals. The dashed black lines indicate mortalities if recovery probability were 1; estimates to the left of the dashed lines are impossible.

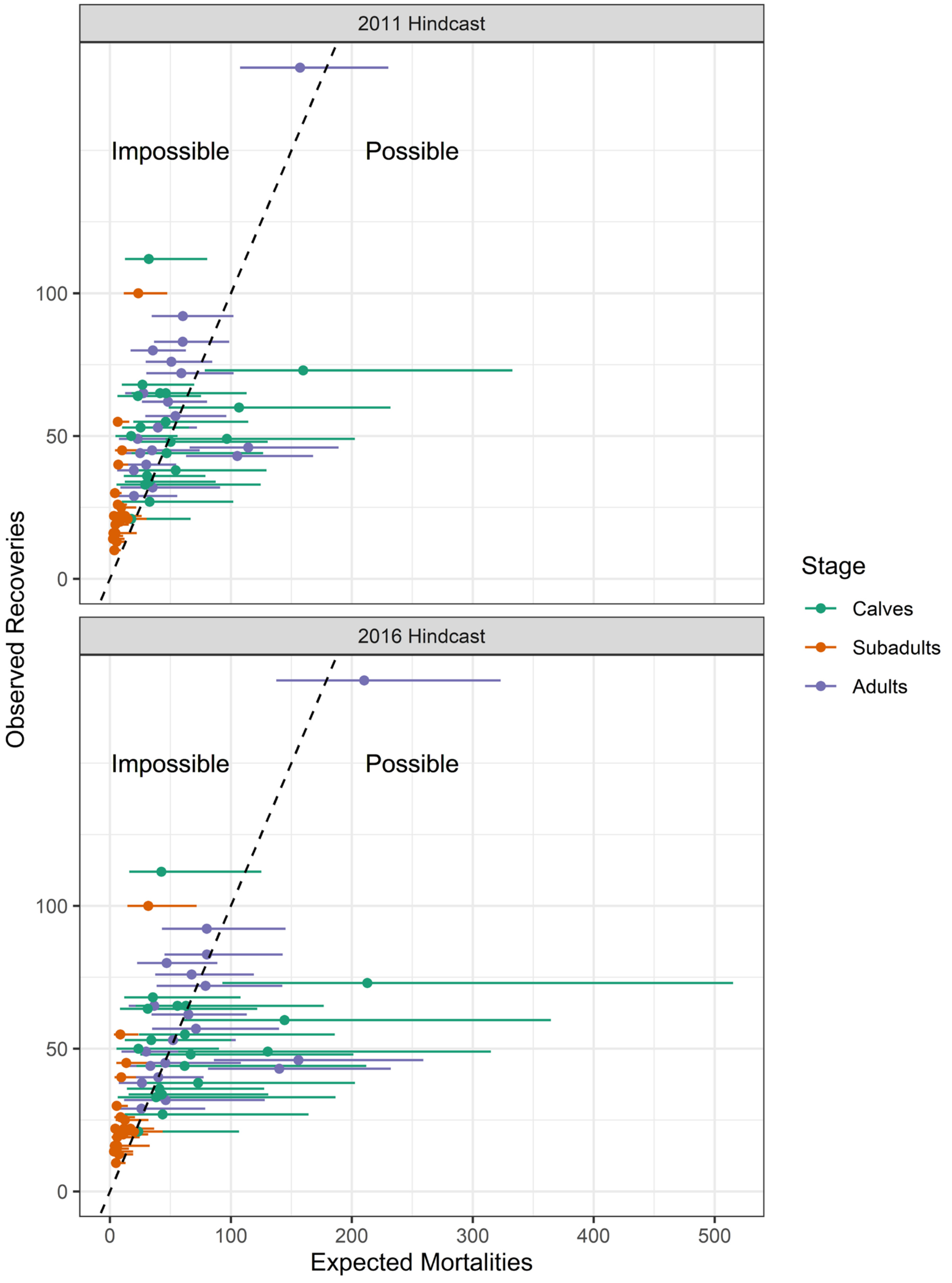

Supplementary Table S1 Assumptions of the integrated population model and its components. Cited references provide more information on models and their assumptions.

Section	Assumption
Integrated model[5–7]	Independence of model components
	All data sets represent the same population of animals
	Validity of individual components and assumptions (or IPM robust to assumption violations)
Adult survival analysis[8]	The population available for capture within a primary period is demographically closed during the period (no births, deaths, emigration or immigration, but model may be robust to violations of this assumption[9])
	Within each group, state, or detection status (previously detected within a primary period or not), each individual has the same probability of detection and state transition
	Identifying marks are retained, recorded correctly, and do not affect survival or behavior
Adult reproductive analysis[10]	For a given state, and for given covariate values, all individuals are equally likely to survive, to transition to another state, to be detected, and to have their current state accurately identified
	Marks do not affect survival or transitions, are not lost, and are recorded correctly
	Each individual acts independently with respect to survival, transition to another state, detection, and state assignment
	For modeling within a primary period, there are no state transitions, and population is closed (no ingress or egress from the set of defined states)
Abundance estimates[11,12]	No double counting of individuals or groups
	Manatees do not move into or out of plots during surveys
	No unmodeled variation in perception probability
	Density/abundance captured by stratified zero-inflated Poisson distribution
	Perception probability for front-seat observers same for individuals and groups of all sizes
	Mean availability probabilities captured by manatee replica experiment
	Statewide manatee diving behavior well estimated with a multisensor digital acoustic tag study
Carcass recoveries	Lengths capture coarse stage
	Year-round carcass recoveries in the southwest region represent animals that spend winter in the southwest region
	Stage and year not interactive for recovery rates
Other	$\gamma_p[t] = \gamma_a[t]$
	There exists for each juvenile age a fixed, though unknown, ratio between its mortality probability for each year and the mortality of adults in the same year
	No temporal variation in γ_4

Supplementary Figure S4. Estimated adult reproductive probabilities from the separate reproductive analysis (IPM prior) and the IPM (IPM posterior) by year. Symbols indicate medians and line segments 95% credible intervals.

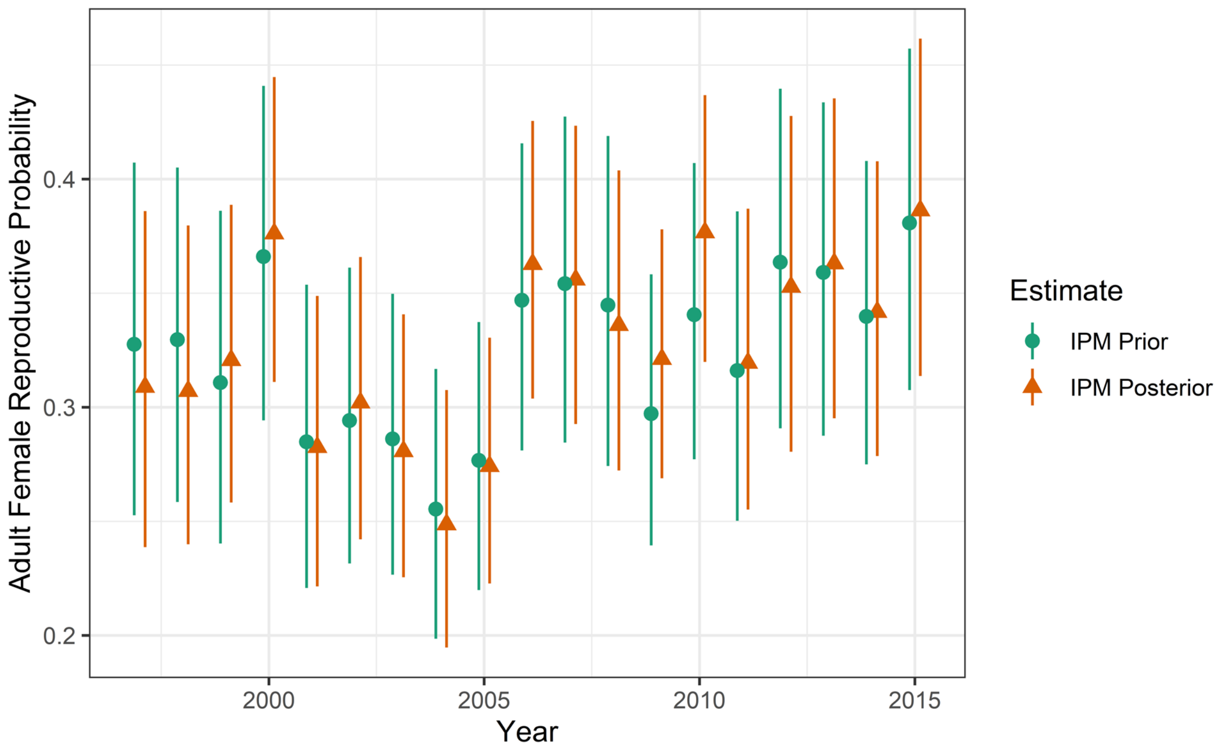

Supplementary Figure S5. Estimated recovery probabilities from the IPM by coarse stage for an average year. Dots indicate medians and line segments 95% credible intervals.

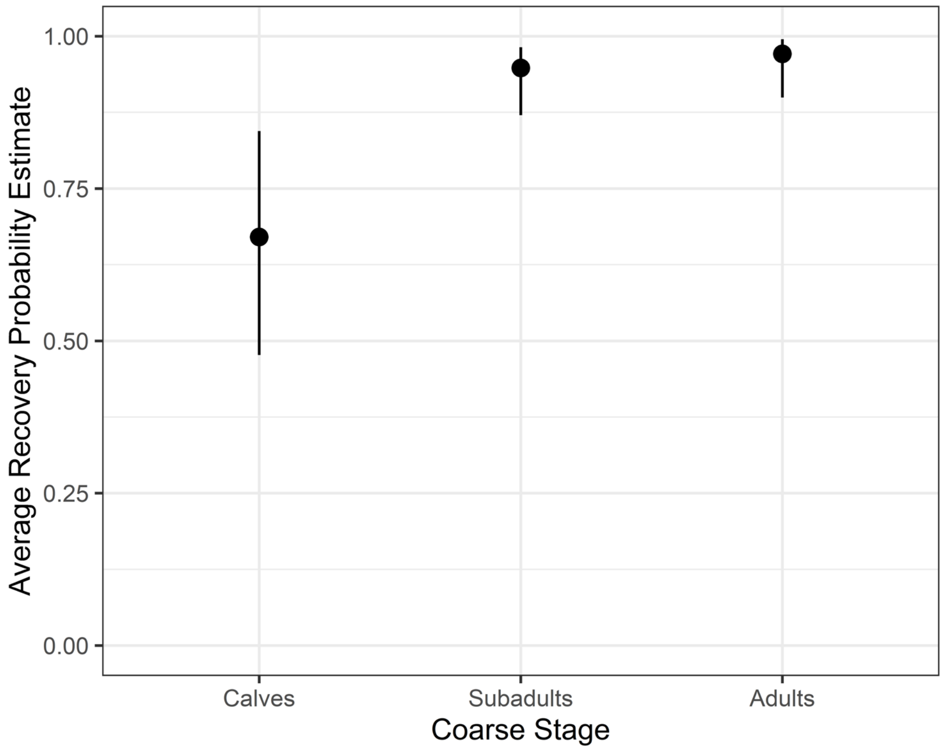

Supplementary Figure S6. Estimated recovery probabilities from the IPM by coarse stage and year. Dots indicate medians and line segments 95% credible intervals. Dotted lines indicate average-year recovery probabilities by coarse stage.

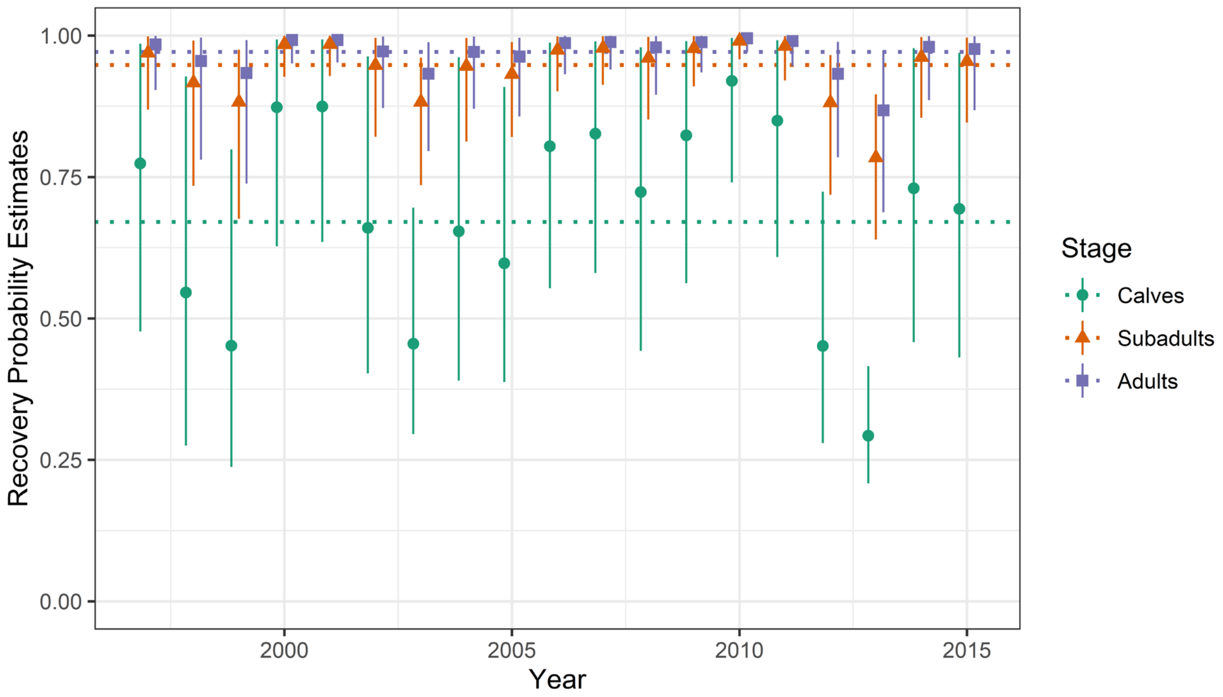

Supplementary Figure S7. Sensitivity analysis of stage structure distribution to initial 1997 stage structure distribution. Symbols indicate medians and line segments 95% credible intervals. Note that the y-axis is scaled differently for each stage class.

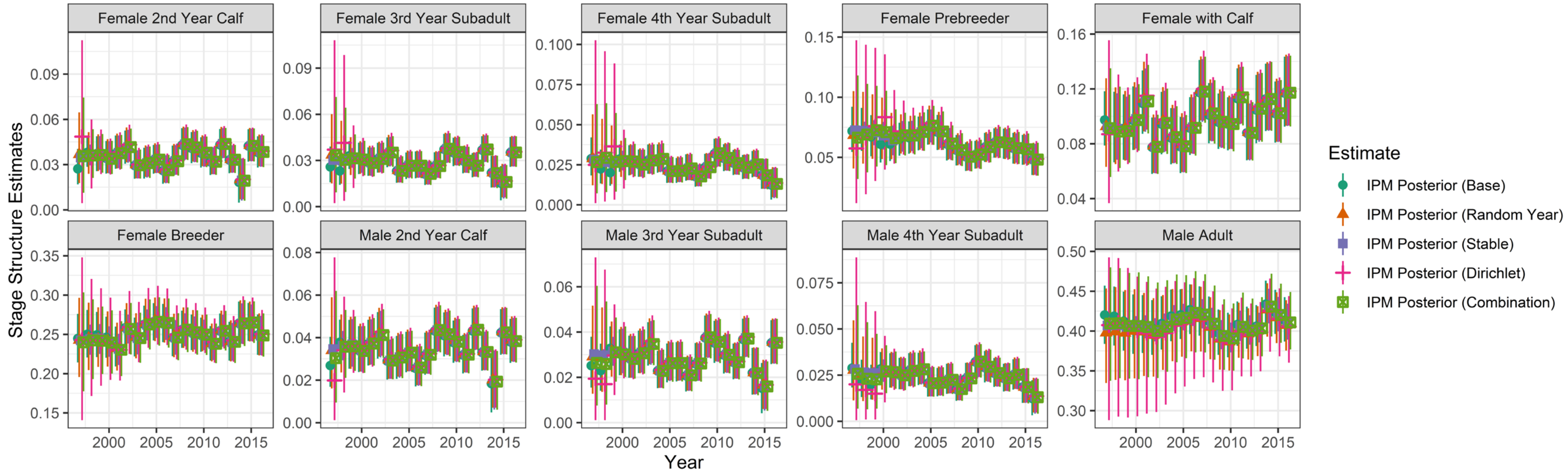

Supplementary Figure S8: Sensitivity analysis of age class structure distribution to initial 1997 stage structure distribution. Symbols indicate medians and line segments 95% credible intervals. Note that the y-axis is scaled differently for each age class.

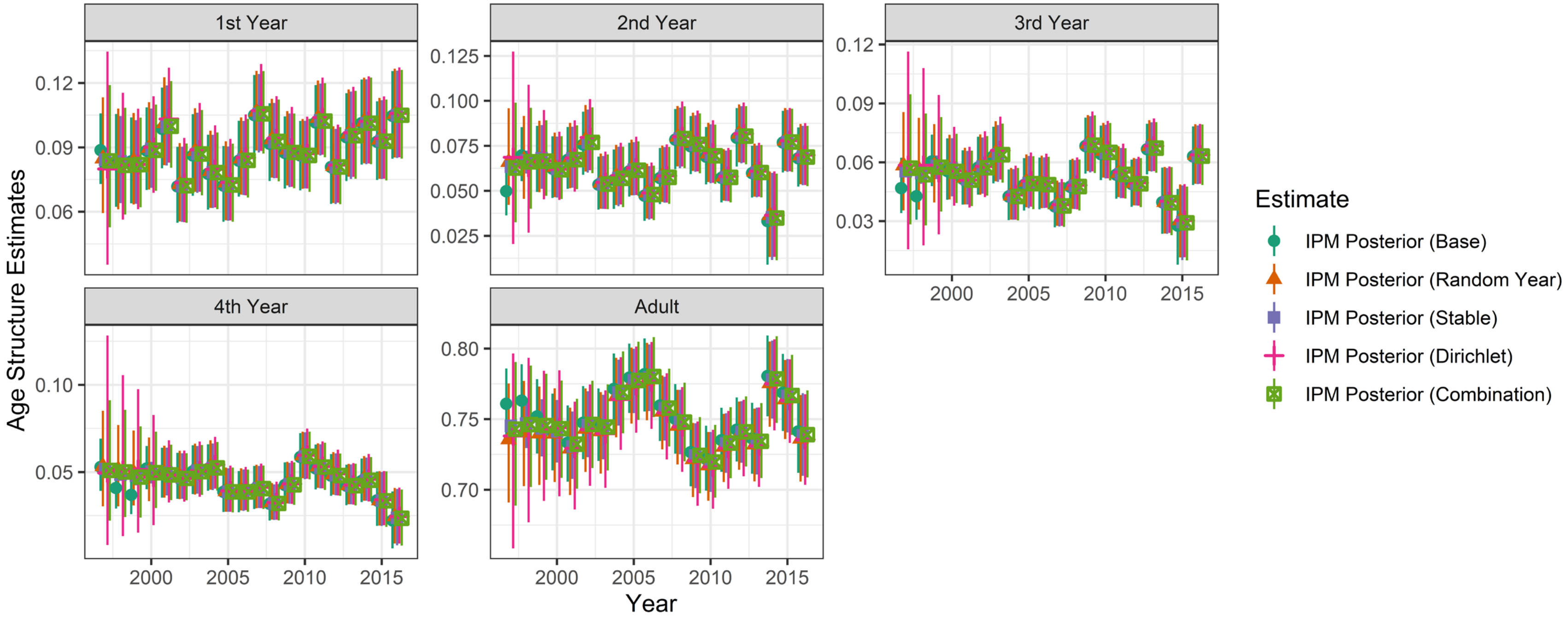

Supplementary Figure S9: Sensitivity analysis of population abundance to initial 1997 stage structure distribution. Symbols indicate medians and line segments 95% credible intervals.

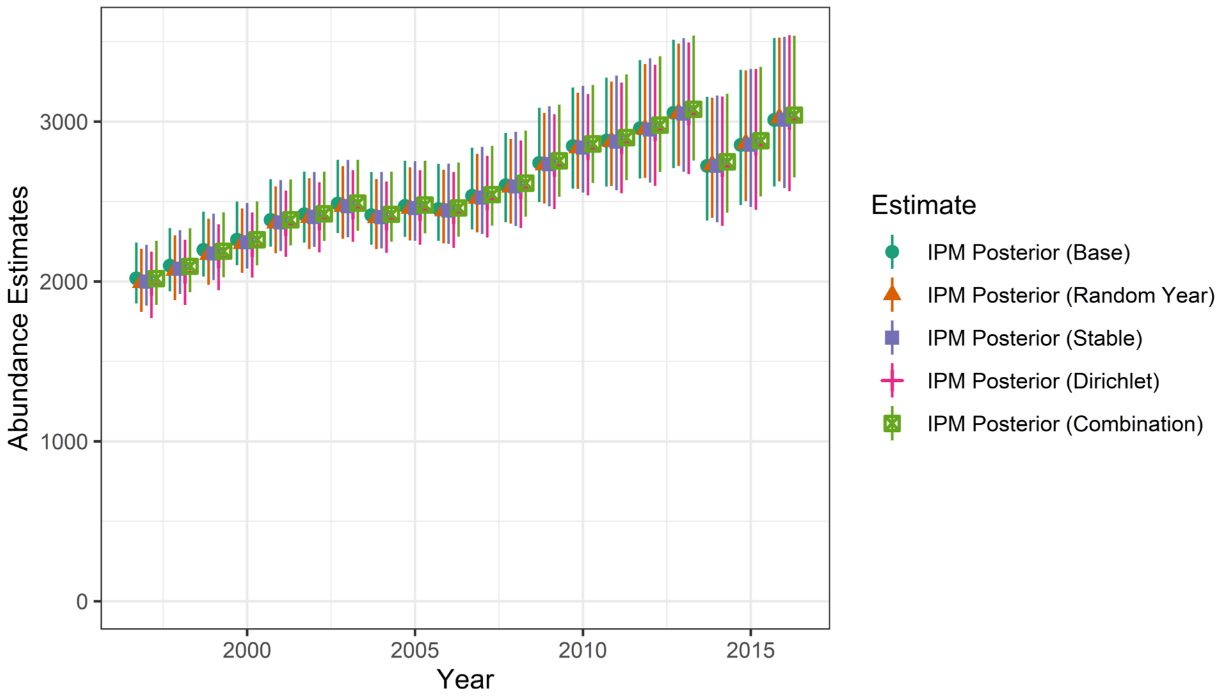

Supplementary Figure S10: Sensitivity analysis of population abundance to initial 1997 abundance distribution. Symbols indicate medians and line segments 95% credible intervals.

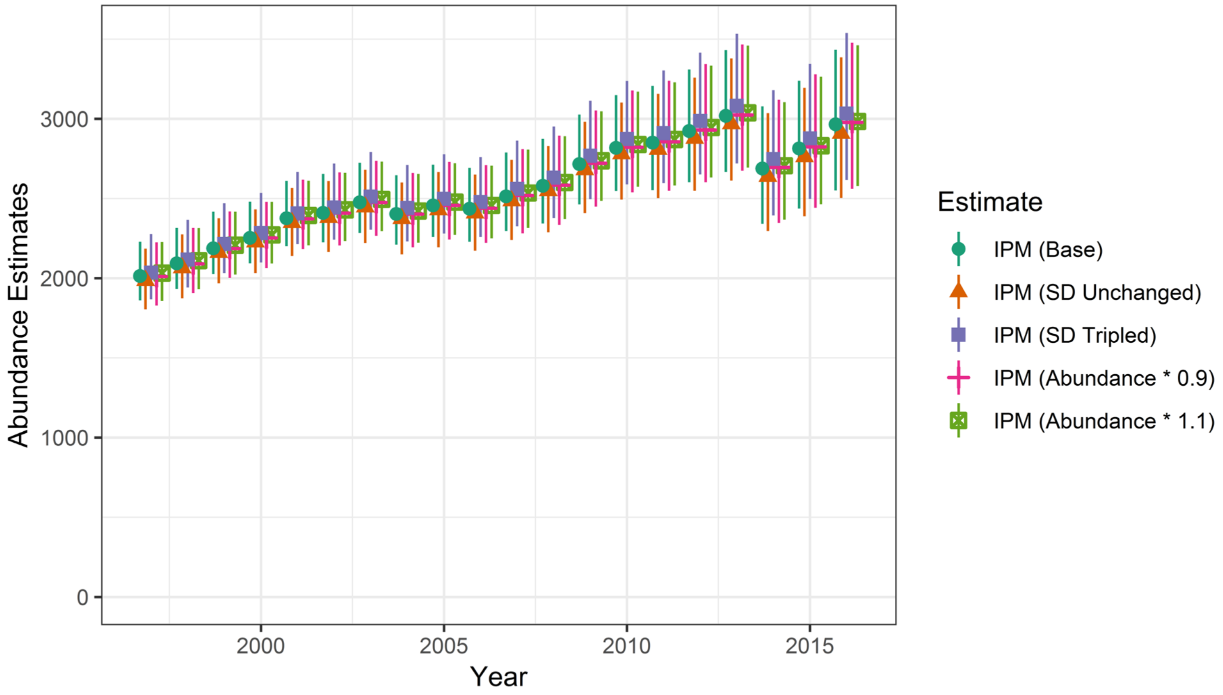

Supplementary Figure S11. Sensitivity analysis of stage structure distribution to initial 1997 abundance distribution. Symbols indicate medians and line segments 95% credible intervals. Note that the y-axis is scaled differently for each stage.

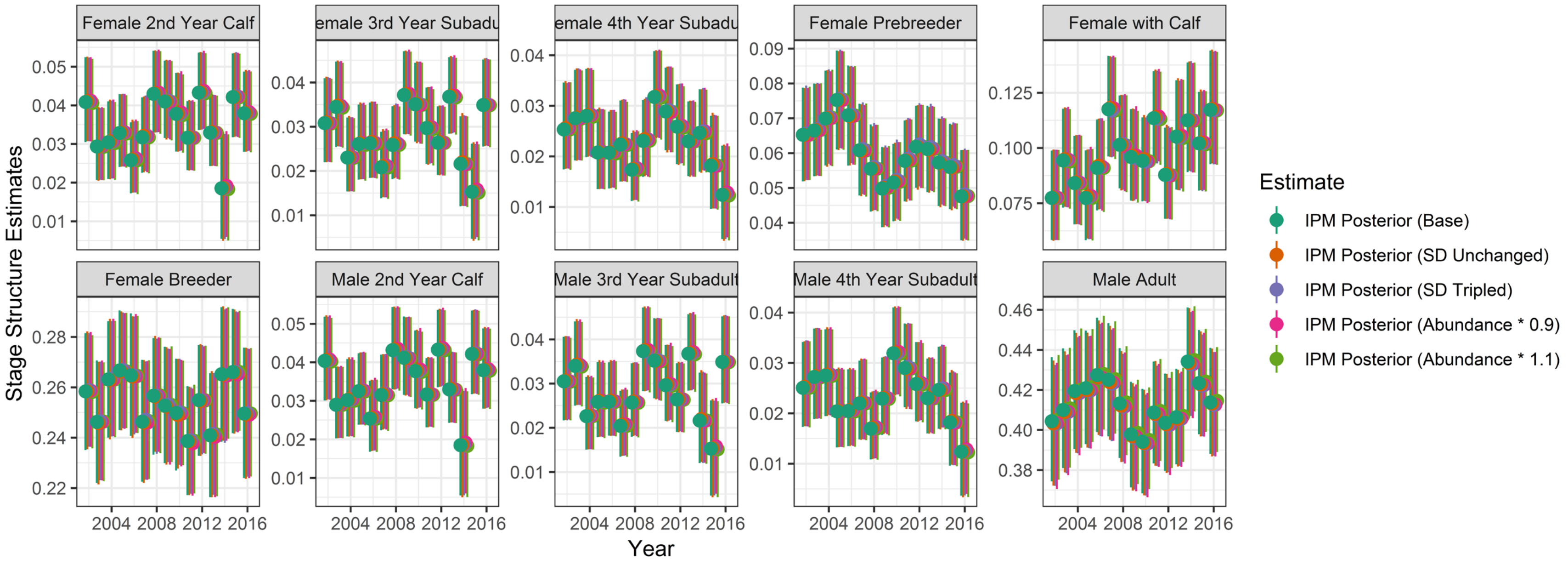

Supplementary Figure S12. Sensitivity analysis of age class structure distribution to initial 1997 abundance distribution. Symbols indicate medians and line segments 95% credible intervals. Note that the y-axis is scaled differently for each stage class.

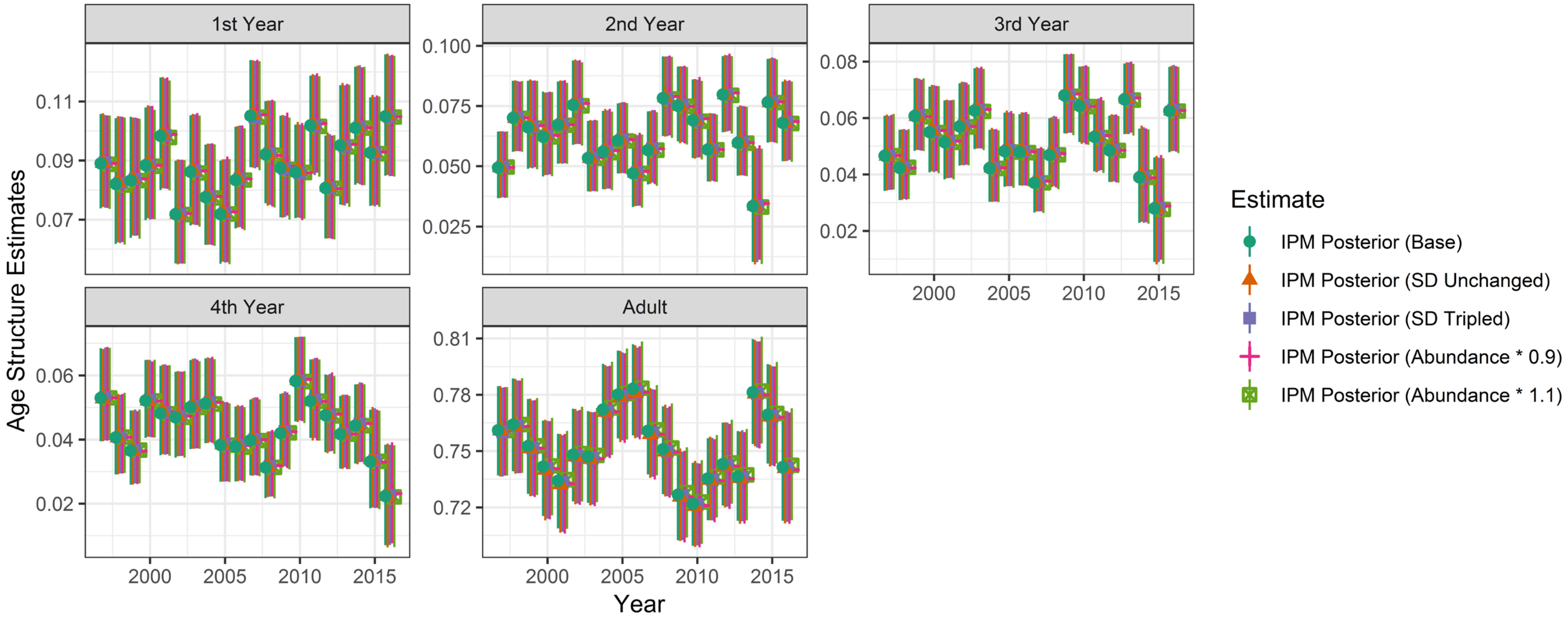

Supplementary Figure S13. Estimated survival probabilities from the IPM by age class and year. Symbols indicate medians and line segments 95% credible intervals.

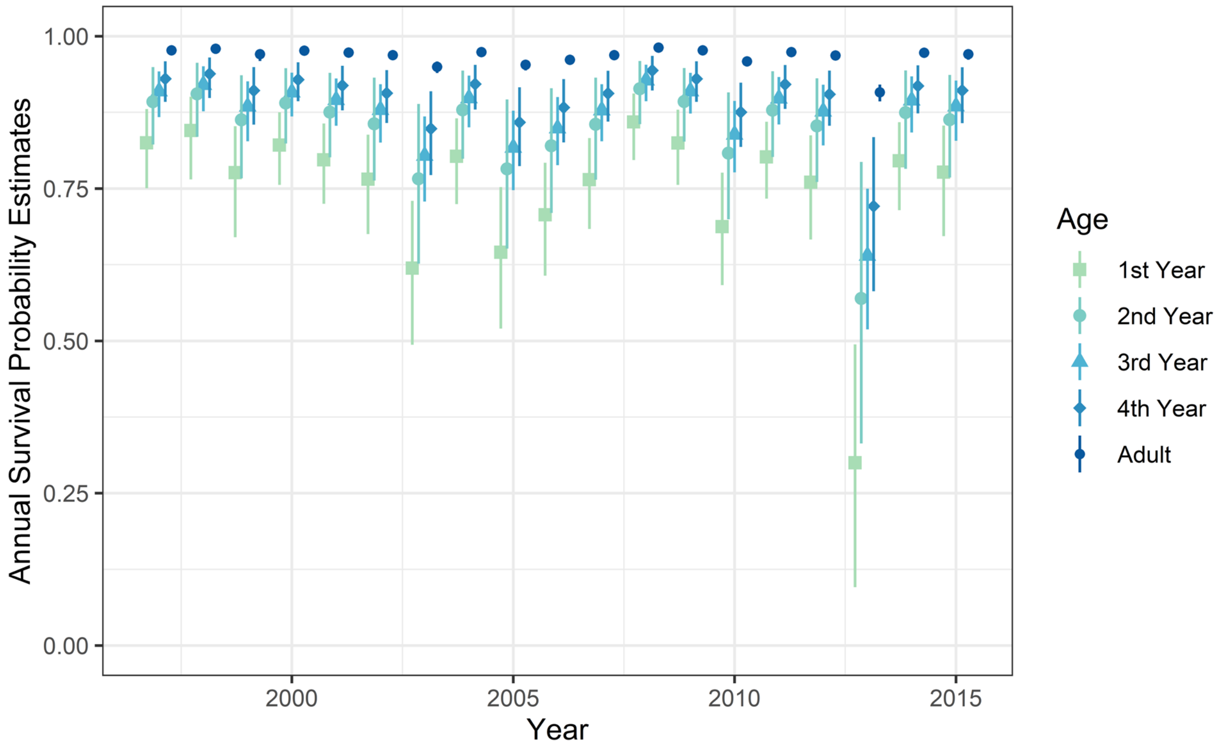

Supplement References

1. Morris, W. F. & Doak, D. F. *Quantitative conservation biology: theory and practice of population viability analysis*. (Sinauer Associates, 2002).
2. Runge, M. C., Sanders-Reed, C. A. & Fonnesbeck, C. J. *A core stochastic population projection model for Florida manatees (*Trichechus manatus latirostris*)*. https://137.227.245.162/resshow/manatee/documents/OFR2007-1082.pdf (U.S. Geological Survey, 2007).
3. Runge, M. C. *et al. Status and threats analysis for the Florida manatee (*Trichechus manatus latirostris*), 2016*. (U.S. Geological Survey, 2017).
4. Brent, R. P. *Algorithms for minimization without derivatives*. (Prentice Hall, 1973).
5. Abadi, F., Gimenez, O., Arlettaz, R. & Schaub, M. An assessment of integrated population models: bias, accuracy, and violation of the assumption of independence. *Ecology* **91**, 7–14 (2010).
6. Weegman, M. D., Arnold, T. W., Clark, R. G. & Schaub, M. Partial and complete dependency among data sets has minimal consequence on estimates from integrated population models. *Ecol. Appl.* e2258 (in press).
7. Riecke, T. V. *et al.* Integrated population models: Model assumptions and inference. *Methods Ecol. Evol.* **10**, 1072–1082 (2019).
8. Kendall, W. L. *et al.* Combining dead recovery, auxiliary observations and robust design data to estimate demographic parameters from marked individuals. *Methods Ecol. Evol.* **4**, 828–835 (2013).
9. Kendall, W. L. Robustness of closed capture–recapture methods to violations of the closure assumption. *Ecology* **80**, 2517–2525 (1999).

10. Kendall, W. L., White, G. C., Hines, J. E., Langtimm, C. A. & Yoshizaki, J. Estimating parameters of hidden Markov models based on marked individuals: use of robust design data. *Ecology* **93**, 913–920 (2012).

11. Martin, J. *et al.* Combining information for monitoring at large spatial scales: first statewide abundance estimate of the Florida manatee. *Biol. Conserv.* **186**, 44–51 (2015).

12. Hostetler, J. A., Edwards, H. H., Martin, J. & Schueller, P. *Updated statewide abundance estimates for the Florida manatee*. https://f50006a.eos-intl.net/F50006A/OPAC/Details/Record.aspx?BibCode=1864664 (Florida Fish and Wildlife Conservation Commission, Fish and Wildlife Research Institute, 2018).
